# [18]F-fluoroethyl-l-tyrosine positron emission tomography for radiotherapy target delineation: Results from a Radiation Oncology credentialing program

**DOI:** 10.1016/j.phro.2024.100568

**Published:** 2024-03-13

**Authors:** Nathaniel Barry, Eng-Siew Koh, Martin A. Ebert, Alisha Moore, Roslyn J. Francis, Pejman Rowshanfarzad, Ghulam Mubashar Hassan, Sweet P. Ng, Michael Back, Benjamin Chua, Mark B. Pinkham, Andrew Pullar, Claire Phillips, Joseph Sia, Peter Gorayski, Hien Le, Suki Gill, Jeremy Croker, Nicholas Bucknell, Catherine Bettington, Farhan Syed, Kylie Jung, Joe Chang, Andrej Bece, Catherine Clark, Mori Wada, Olivia Cook, Angela Whitehead, Alana Rossi, Andrew Grose, Andrew M. Scott

**Affiliations:** a School of Physics, Mathematics and Computing, University of Western Australia, Crawley, WA, Australia; b South Western Sydney Clinical School, University of New South Wales, Australia; c Department of Radiation Oncology, Sir Charles Gairdner Hospital, Nedlands, WA, Australia; d Trans Tasman Radiation Oncology Group (TROG) Cancer Research, Newcastle, NSW Australia; e Department of Nuclear Medicine, Sir Charles Gairdner Hospital, Nedlands, WA, Australia; f Department of Radiation Oncology, Austin Health, Heidelberg, VIC, Australia; g Department of Radiation Oncology, Royal North Shore Hospital, Sydney, NSW, Australia; h Department of Radiation Oncology, Royal Brisbane Womens Hospital, Brisbane, QLD, Australia; i Department of Radiation Oncology, Princess Alexandra Hospital, Brisbane, QLD, Australia; j Department of Radiation Oncology, Peter MacCallum Cancer Centre, VIC, Australia; k Department of Radiation Oncology, Royal Adelaide Hospital, Adelaide, SA, Australia; l Department of Radiation Oncology, The Canberra Hospital, Canberra, ACT, Australia; m Department of Radiation Oncology, St George Hospital, Kogarah, NSW, Australia; n Australian Centre for Quantitative Imaging, Medical School, University of Western Australia, Crawley, WA, Australia; o Department of Molecular Imaging and Therapy, Austin Health, and University of Melbourne, Melbourne, VIC, Australia; p Olivia Newton-John Cancer Research Institute, and School of Cancer Medicine La Trobe University, Melbourne, VIC, Australia; q Centre for Advanced Technologies in Cancer Research (CATCR), Perth, WA, Australia

**Keywords:** FET PET, Glioblastoma, Treatment planning, Credentialing, Clinical trials

## Abstract

**Background and purpose:**

The [18]F-fluoroethyl-l-tyrosine (FET) PET in Glioblastoma (FIG) study is an Australian prospective, multi-centre trial evaluating FET PET for newly diagnosed glioblastoma management. The Radiation Oncology credentialing program aimed to assess the feasibility in Radiation Oncologist (RO) derivation of standard-of-care target volumes (TV_MR_) and hybrid target volumes (TV_MR+FET_) incorporating pre-defined FET PET biological tumour volumes (BTVs).

**Materials and methods:**

Central review and analysis of TV_MR_ and TV_MR+FET_ was undertaken across three benchmarking cases. BTVs were pre-defined by a sole nuclear medicine expert. Intraclass correlation coefficient (ICC) confidence intervals (CIs) evaluated volume agreement. RO contour spatial and boundary agreement were evaluated (Dice similarity coefficient [DSC], Jaccard index [JAC], overlap volume [OV], Hausdorff distance [HD] and mean absolute surface distance [MASD]). Dose plan generation (one case per site) was assessed.

**Results:**

Data from 19 ROs across 10 trial sites (54 initial submissions, 8 resubmissions requested, 4 conditional passes) was assessed with an initial pass rate of 77.8 %; all resubmissions passed. TV_MR+FET_ were significantly larger than TV_MR_ (p < 0.001) for all cases. RO gross tumour volume (GTV) agreement was moderate-to-excellent for GTV_MR_ (ICC = 0.910; 95 % CI, 0.708–0.997) and good-to-excellent for GTV_MR+FET_ (ICC = 0.965; 95 % CI, 0.871–0.999). GTV_MR+FET_ showed greater spatial overlap and boundary agreement compared to GTV_MR_. For the clinical target volume (CTV), CTV_MR+FET_ showed lower average boundary agreement versus CTV_MR_ (MASD: 1.73 mm vs. 1.61 mm, p = 0.042). All sites passed the planning exercise.

**Conclusions:**

The credentialing program demonstrated feasibility in successful credentialing of 19 ROs across 10 sites, increasing national expertise in TV_MR+FET_ delineation.

## Highlights

• 19 Radiation Oncologists (ROs) were credentialed for [18]F-fluoroethyl-l-tyrosine (FET) positron emission tomography (PET) in Glioblastoma (FIG) study.
• ROs integrated FET PET and magnetic resonance imaging (MRI) data to derive hybrid target volumes on three cases.
• Across 10 FIG trial sites, the initial pass rate was 77.8%. All resubmissions passed.
• Hybrid gross tumour volume had greater volume, spatial and boundary agreement than MRI-based gross tumour volume.

## 1 Introduction

Glioblastoma is the most common adult primary brain malignancy with a poor prognosis. Conventional treatment is maximal safe resection followed by adjuvant radiotherapy (RT) with concurrent and adjuvant Temozolomide chemotherapy [1], [2], [3]. MRI utilising T1-weighted pre- and post-contrast (T1c), T2-weighted, and fluid attenuated inversion recovery (FLAIR) sequences are routinely used to delineate RT target volumes (TVs). Recent ESTRO-EANO guidelines define gross tumour volume (GTV) as the post-operative resection cavity plus any residual enhancing tumour on T1c, with expansion to clinical target volume (CTV) using a margin of 15 mm. Furthermore, changes on T2/FLAIR that are felt to reflect non-enhancing tumour should be incorporated into the CTV [4]. The role of perfusion, diffusion and MR spectroscopy in target volume definition are currently not well defined and therefore not routine [4], [5], [6], [7].

[18]F-fluorodeoxyglucose (FDG) PET is an established approach for imaging tumours; however, low FDG tumour-to-background ratios (TBRs) can lead to challenges in delineating brain tumour extent due to high background uptake in grey matter. Comparatively, amino acid tracers exhibit higher TBRs and do not have such limitation [8]. Clinical studies have focused on the use of [18]F-fluoroethyl-l-tyrosine (FET) PET post-RT for differentiation of treatment-related changes from tumour recurrence [9], [10], [11]. FET PET may also have a role in delineating tumour extent in combination with MRI in the immediate post-surgical and pre-RT setting [12], [13], [14], [15], [16], [17]. To date, evidence for the utility of FET PET has been predominantly from smaller, single centre studies.

The [18]F-fluoroethyl-l-tyrosine (FET) PET in Glioblastoma (FIG) study is an Australian prospective, multi-centre trial evaluating the impact of FET PET on the management of adult patients with newly diagnosed glioblastoma [18]. Participants undergo FET PET imaging pre-chemoradiotherapy (FET1), one-month post-chemoradiotherapy (FET2) and at suspected progression (FET3). Successful completion of Radiation Oncologist (RO) and Nuclear Medicine Physician (NMP) credentialing was required of all participating centres before participant enrolment, with the results of NMP credentialing published recently [19]. In the FIG study, radiotherapy TVs for participants are delineated as per standard-of-care, with hybrid volumes derived post-hoc using both FET PET and MRI. The RO credentialing program focused on incorporating a FET PET biological tumour volume (BTV), along with standard MRI information, to delineate hybrid TVs compared to MRI alone. The feasibility of this process was evaluated across multiple ROs and 10 trial sites across Australia. Analysis of the resulting data include: a summary of expert central reviews, quantitative pairwise analysis of RO contours and comparison of standard and hybrid TVs. Additionally, per study site, central review of standard-of-care dosimetry plans and constraints was conducted, with analysis of any dose variability presented.

## 2 Material and methods

Three benchmarking cases with de-identified glioblastoma patient imaging, taken prior to RT, were chosen for credentialing (FET1CASE1, FET1CASE2, FET1CASE3, respectively). Local ethics approval was obtained for use of these three cases. Each patient dataset contained a planning CT (pCT), T1c, T2-weighted or T2 FLAIR as well as FET PET dynamic and static images. These cases represented distinct clinical scenarios with gadolinium enhancing disease (Table 1). Further detail on MRI acquisition parameters can be found in Supplementary Table 1.

**Table 1:** Patient characteristics and biological tumour volume size.

	FET1CASE1	FET1CASE2^a^	FET1CASE3
Sex, age	F, 58	M, 67	F, 60
Lesion location	Right frontoparietal	Right temporal	Left frontoparietal

Surgery	Partial resection	Gross total resection	Resection^b^
Prescribed RT (Gy/#fractions)	60/30	60/30	40.05/15

Pathology			
MGMT methylation	Unmethylated	Methylated	Unknown
IDH1 mutation	Wildtype	Wildtype	Unknown

BTV size (cc)	31.7	69.5	13.1

M = Male, F = Female, RT Radiotherapy.

MGMT O(6)-methylguanine-DNA methyltransferase.

IDH Isocitrate dehydrogenase.

BTV Biological tumour volume.

a Additionally used as the planning case.

b Extent not specified.

### 2.1 FET PET acquisition and contouring

Benchmarking cases were obtained from three different glioblastoma patients treated at Sir Charles Gairdner Hospital, Nedlands, Western Australia, from a previous study (Human Research Ethics Committee approved study 2014-004). Patients fasted for a minimum of 4 h prior to imaging. FET PET scans were acquired following intravenous administration of 200 MBq of FET, on a Biograph 16 PET/CT (Siemens CTI Inc, Knoxville, TN). A low dose CT was performed for attenuation correction. A 30-minute dynamic acquisition followed with the final static image consisting of summed PET data 20–30 min post-injection of tracer. Dead time, attenuation, scatter, decay, and random corrections were applied, along with detector normalisation. Iterative reconstruction for the FET1 cases was performed with a point-spread function applied (TrueX, 3i24s, matrix = 168 × 168, zoom factor 2, 4 mm Gaussian post-filter). NMP delineation of FET PET was performed using a MiM workflow developed for the FIG study (MiM Encore version 7.0**,** MiM Software Inc, Cleveland OH). BTV delineation was performed by a sole expert NMP (RJF), using the following semi-automatic procedure: a crescent-shaped volume of interest (VOI), including grey and white matter, was placed in the hemisphere contralateral to the suspected lesion to assess mean background uptake, using T1c for anatomic reference [20]. The BTV was defined using a 1.6 TBR threshold on a spherical VOI placed around the suspected tumour [21]. The BTV was manually adjusted to remove any obvious non-tumour structures.

### 2.2 RO credentialing workflow

Each RO downloaded the credentialing cases into the treatment planning system (TPS) routinely used at their study site and co-registered images as per standard contouring. Target volume and organ at risk (OAR) contouring guidelines were provided in the FIG trial radiotherapy and quality assurance (RTQA) manual (Supplementary Tables 2–4). Critical OARs were the brainstem and optic chiasm. The optic nerves, retinas, eyes, and lenses were requested contours for FET1CASE1 only [4], [22]. ROs were instructed to not review or use the FET PET data for delineation of standard-of-care TVs: GTV_MR_, CTV_MR_ with 15 mm margin clipped at anatomical barriers (e.g., tentorium, meninges), and planning target volume (PTV_MR_) with 3 mm margin [4]. These TVs were then “turned off” prior to introducing and registering FET PET data to CT/MRI, which included the BTV, thus mimicking the “blinding” component built into the FIG trial. ROs received the same BTV for each case and were instructed to incorporate it into hybrid MR+FET-derived TVs, as stated in the RTQA manual: GTV_MR+FET_, CTV_MR+FET_ with 10–15 mm margin, and PTV_MR+FET_ with 3 mm margin, without reference to the original standard-of-care TVs [4], [23]. All structures were registered to pCT. Additionally, one standard-of-care pCT, with contoured TVs and OARs, was derived from FET1CASE2 for dose plan generation. A review per study site was conducted to evaluate technique and dose goals (Supplementary Table 5). Completed credentialing cases were reviewed via the TROG (Trans-Tasman Radiation Oncology Group) server by one of three experts (ESK, BC, MB) to assess protocol compliance. Delineating these structures was assessed as acceptable, minor or major violations, or missing. Violation reasons were documented with the incidence and reasons for resubmission recorded.

### 2.3 Statistical analysis

Descriptive statistics are reported as mean/standard deviation and median/range. All contours in DICOM RTStruct format were converted to binary mask using Plastimatch.^1^ Spatial and boundary agreement between two segmentations was assessed using the Dice similarity coefficient (DSC), Jaccard index (JAC), overlap volume (OV), Hausdorff distance (HD) and mean absolute surface distance (MASD) [24]. All metrics were calculated using PlatiPy^2^ [25]. For each case, pairwise comparison of RO contours was undertaken. Each ROs pair of standard and hybrid TVs were also directly compared (GTV_paired_, CTV_paired_, PTV_paired_). The intraclass correlation coefficient (ICC) using a two-way mixed model (absolute agreement, single rater/measurement) assessed volume agreement [26], [27]. The ICC was interpreted based on recommendations by Koo and Li [28], calculated using the ‘psych’ package from R combined with the rpy2 interface.^3^ Dose plan variability was assessed using D_2%_, D_50%_, D_98%_, V_95%_, conformity index and homogeneity index. Definitions of all metrics included can be found in the Supplementary material. The Wilcoxon signed-rank test assessed differences between metrics evaluated on standard and hybrid TVs. Bonferroni adjustment for multiple testing was applied, with a p-value < 0.05 classed as significant.

## 3 Results

Ten FIG study sites participated in the credentialing program with a total of 19 ROs submitting data for review. ROs had a range of neuro-oncology expertise (n = 3, <5 years; n = 7, 5–9 years; n = 9; 10+ years) and prior familiarity with FET PET (n = 9, none; n = 7, minimal; n = 0, moderate; n = 3, significant). In all centres, except for one, two ROs per site underwent FIG trial credentialing. There were 19 sets of contours received for FET1CASE1 and FET1CASE2, and 16 for FET1CASE3 (three sets of contours not received), respectively.

### 3.1 Credentialing case reviews

**Fig. 1:**
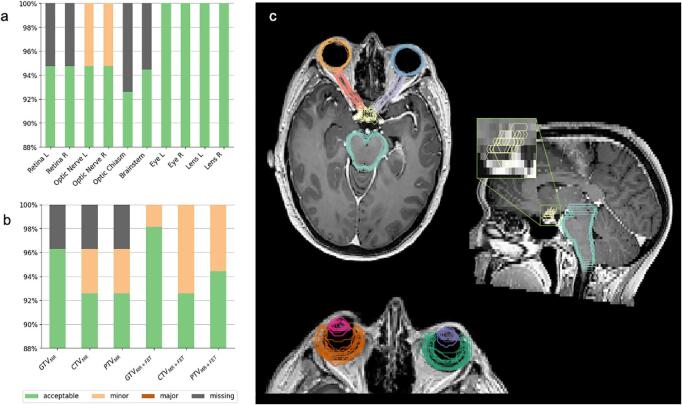
Summary of the initial reports of organ at risk (OAR) and target volume (TV) contours generated as part of credentialing of each Radiation Oncologist. Stacked bar charts illustrate delineation violations of OARs (a), MR-derived and MR+FET-derived TVs (b) with respective OARs shown on the FET1CASE1 axial and sagittal views of the contrast-enhanced MRI (c).

Case reviews were conducted on 54 initial submissions, with eight additional requested resubmissions (n = 6, missing contour(s); n = 2, contour data not registered to pCT) and four conditional passes (n = 3, image registration misalignment; n = 1, missing contour(s)), where the observer was provided feedback to be followed for the prospective phase. This resulted in an initial 77.8 % pass rate. The initial reports are summarised in Fig. 1. All resubmissions were subsequently passed, and all missing TVs were received for quantitative analysis. All ten dosimetry plans (one per site) utilising four different TPSs (Supplementary Table 6), were within dose constraints, with no resubmissions required. Six reports contained seven minor violations only (technique, n = 3; dose, n = 4).

### 3.2 Comparison of standard versus hybrid target volumes

**Fig. 2:**
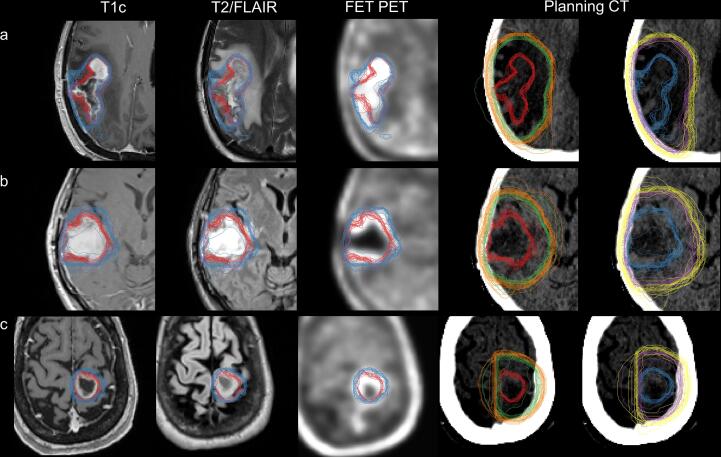
An overview of all Radiation Oncology target volume (TV) delineations on axial imaging. Each row represents the benchmarking cases FET1CASE1 (a), FET1CASE2 (b), and FET1CASE3 (c). Standard GTV_MR_ (red), CTV_MR_ (green), PTV_MR_ (orange), and hybrid GTV_MR+FET_ (blue), CTV_MR+FET_ (purple), PTV_MR+FET_ (yellow) are shown. For comparison, GTV_MR_ and GTV_MR+FET_ are shown together with T1c, T2/FLAIR, and FET PET with all images co-registered to each cases respective planning CT where all TV_MR_ and TV_MR+FET_ are displayed separately. (For interpretation of the references to colour in this figure legend, the reader is referred to the web version of this article.)

Hybrid TVs were significantly larger than standard TVs (*p* < 0.001) for all cases (Table 2). Observer TVs are illustrated in Fig. 2. The ICC was calculated from ROs that had completed contouring on all three cases (16/19 observers), which was moderate to excellent (ICC = 0.910; 95 % CI, 0.708–0.997) for GTV_MR_ and good to excellent (ICC = 0.965; 95 % CI, 0.871–0.999) for GTV_MR+FET_. Further results can be found in Supplementary Table 7.

**Table 2:** Mean and standard deviation of target volumes and pairwise analysis metrics for each case and across all cases.

		GTV_MR_	GTV_MR+FET_	p-value	CTV_MR_	CTV_MR+FET_	p-value	PTV_MR_	PTV_MR+FET_	p-value
	Volume (cc)									
FET1CASE1		23.40 ± 4.40	38.43 ± 4.06	**< 0.001**	163.03 ± 14.49	194.98 ± 17.67	**< 0.001**	219.91 ± 19.40	264.18 ± 24.49	**< 0.001**
FET1CASE2		51.09 ± 10.04	82.46 ± 10.23	**< 0.001**	203.24 ± 31.15	256.32 ± 28.46	**< 0.001**	268.98 ± 41.61	333.55 ± 41.30	**< 0.001**
FET1CASE3		8.11 ± 0.91	16.43 ± 1.97	**< 0.001**	58.61 ± 8.02	80.23 ± 11.51	**< 0.001**	86.59 ± 10.43	115.39 ± 16.37	**< 0.001**

	Pairwise									
FET1CASE1										
	DSC	0.81 ± 0.06	0.84 ± 0.04	**< 0.001**	0.92 ± 0.04	0.91 ± 0.04	0.091	0.93 ± 0.03	0.91 ± 0.04	0.024
	JAC	0.69 ± 0.09	0.73 ± 0.06	**< 0.001**	0.85 ± 0.06	0.83 ± 0.07	0.083	0.86 ± 0.05	0.84 ± 0.06	0.020
	OV	0.89 ± 0.06	0.89 ± 0.04	0.958	0.96 ± 0.03	0.96 ± 0.02	0.015	0.97 ± 0.02	0.97 ± 0.02	0.007
	HD (mm)	10.46 ± 6.66	8.57 ± 3.18	**< 0.001**	11.08 ± 4.92	9.01 ± 3.01	**< 0.001**	11.42 ± 4.56	9.15 ± 3.35	**< 0.001**
	MASD (mm)	1.11 ± 0.69	1.10 ± 0.36	0.222	1.24 ± 0.72	1.48 ± 0.82	0.011	1.26 ± 0.60	1.61 ± 0.85	**< 0.001**

FET1CASE2										
	DSC	0.84 ± 0.06	0.86 ± 0.06	**< 0.001**	0.89 ± 0.05	0.90 ± 0.04	0.002	0.89 ± 0.05	0.90 ± 0.04	**< 0.001**
	JAC	0.72 ± 0.08	0.76 ± 0.08	**< 0.001**	0.80 ± 0.08	0.82 ± 0.06	0.002	0.81 ± 0.08	0.82 ± 0.07	**< 0.001**
	OV	0.93 ± 0.04	0.93 ± 0.04	0.231	0.96 ± 0.03	0.96 ± 0.03	0.153	0.97 ± 0.03	0.97 ± 0.03	0.301
	HD (mm)	17.25 ± 7.94	14.33 ± 6.26	**< 0.001**	14.74 ± 5.53	13.26 ± 5.29	**< 0.001**	14.93 ± 5.62	13.55 ± 5.43	**< 0.001**
	MASD (mm)	2.07 ± 1.06	1.69 ± 0.69	**< 0.001**	2.06 ± 1.16	1.98 ± 0.86	0.325	2.27 ± 1.27	2.19 ± 1.05	0.142

FET1CASE3										
	DSC	0.87 ± 0.06	0.86 ± 0.07	0.971	0.88 ± 0.06	0.87 ± 0.07	0.791	0.89 ± 0.05	0.88 ± 0.06	0.170
	JAC	0.77 ± 0.09	0.76 ± 0.10	0.935	0.78 ± 0.09	0.78 ± 0.10	0.890	0.80 ± 0.08	0.79 ± 0.09	0.180
	OV	0.91 ± 0.05	0.91 ± 0.05	0.245	0.94 ± 0.04	0.95 ± 0.04	0.085	0.95 ± 0.04	0.96 ± 0.04	0.021
	HD (mm)	4.44 ± 2.59	4.96 ± 1.60	0.002	8.83 ± 4.38	9.77 ± 4.11	0.003	8.99 ± 4.20	10.78 ± 4.16	**< 0.001**
	MASD (mm)	0.86 ± 0.46	1.06 ± 0.56	0.014	1.50 ± 0.83	1.72 ± 0.96	0.013	1.65 ± 0.90	1.95 ± 1.03	0.003

ALL										
	DSC	0.83 ± 0.06	0.85 ± 0.05	**< 0.001**	0.90 ± 0.05	0.89 ± 0.05	0.426	0.90 ± 0.05	0.90 ± 0.05	0.861
	JAC	0.72 ± 0.09	0.75 ± 0.08	**< 0.001**	0.81 ± 0.08	0.81 ± 0.08	0.422	0.83 ± 0.08	0.82 ± 0.08	0.912
	OV	0.91 ± 0.05	0.91 ± 0.04	0.887	0.96 ± 0.03	0.96 ± 0.03	0.174	0.97 ± 0.03	0.96 ± 0.03	0.358
	HD (mm)	11.41 ± 8.19	9.76 ± 5.76	**< 0.001**	11.85 ± 5.56	10.78 ± 4.67	**< 0.001**	12.09 ± 5.44	11.2 ± 4.82	**< 0.001**
	MASD (mm)	1.40 ± 0.96	1.31 ± 0.62	0.066	1.61 ± 1.00	1.73 ± 0.90	0.042	1.74 ± 1.06	1.91 ± 1.01	0.005

Significant comparisons adjusted for multiple testing are in bold (p < 0.001).

DSC Dice Similarity Coefficient, JAC Jaccard Index, OV Overlap Volume, HD Hausdorff Distance, MASD Mean Absolute Surface Distance.

### 3.3 Pairwise analysis of contour agreement

**Fig. 3:**
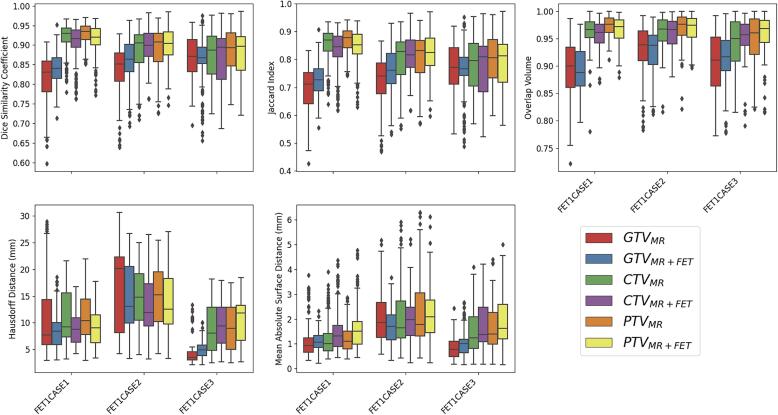
Boxplots showing the distribution of metrics calculated in pairwise comparisons of observer target volume delineations grouped by each benchmarking case. MR-derived and MR+FET-derived boxplots are shown side-by-side for comparison.

The brainstem and eyes exhibited the highest spatial agreement whereas the optic chiasm exhibited the least, sometimes not overlapping at all. Spatial and boundary metrics for all TVs are reported in Table 2. Overall, GTV_MR_ exhibited lower spatial overlap compared to GTV_MR+FET_ (DSC, 0.83 vs. 0.85, *p* < 0.001; JAC, 0.72 vs. 0.75, *p* < 0.001) and lower boundary agreement (HD, 11.41 mm vs. 9.76 mm, *p* < 0.001; MASD, 1.40 mm vs. 1.31 mm, *p* = 0.066), with equal OV (0.91 vs. 0.91, p = 0.887). Distribution of these metrics, by each case, is shown in Fig. 3. Differences in MASD for GTV_MR_ and GTV_MR+FET_ was only significant in FET1CASE2 (2.07 mm vs. 1.69 mm, *p* < 0.001) which had the largest BTV to incorporate. CTV_MR_ and CTV_MR+FET_ spatial overlap was similar (DSC, 0.90 vs. 0.89, *p* = 0.426; JAC 0.81 vs. 0.81, *p* = 0.422; OV 0.96 vs. 0.96, *p* = 0.174). HD was larger for CTV_MR_ compared to CTV_MR+FET_ (11.85 mm vs. 10.78 mm, *p* < 0.001). However, MASD was lower on average for CTV_MR_ compared to CTV_MR+FET_ (1.61 mm vs. 1.73 mm, *p* = 0.042). This trend was also observed for PTV_MR_ and PTV_MR+FET_. Further information can be found in Supplementary Tables 8–12 and Supplementary Figs. 1-5.

### 3.4 Analysis of each RO’s pair of standard and hybrid contours

Comparison results for GTV_paired_, CTV_paired_, and PTV_paired_ is shown in Table 3. Overall, GTV_paired_ spatial overlap was moderate (DSC, 0.71; JAC, 0.55) which increased for CTV_paired_ (DSC, 0.85; JAC, 0.75) and PTV_paired_ (DSC, 0.87; JAC, 0.77), likely reflective of the concentric margin expansion. GTV_paired_ MASD was typically 2–3.5 mm with some comparisons up to almost 5 mm. Average HD measured 12.75 mm, which exceeded 15 mm in some instances (over 20 mm for FET1CASE2). These boundary differences generally propagated to CTV_paired_ (HD, 12.26 mm; MASD, 2.47 mm) and PTV_paired_ (HD, 12.33 mm; MASD, 2.57 mm) upon expansion.

**Table 3:** Intra-observer clinical-to-hybrid comparison. The below metrics were calculated between each Radiation Oncologist’s paired MR-derived target volumes and their respective MR+FET-derived target volumes.

Case		GTV_paired_	CTV_paired_	PTV_paired_
FET1CASE1				
	DSC	0.72 ± 0.06	0.87 ± 0.03	0.89 ± 0.03
	JAC	0.57 ± 0.08	0.78 ± 0.05	0.80 ± 0.05
	OV	0.96 ± 0.06	0.97 ± 0.04	0.98 ± 0.02
	HD (mm)	15.54 ± 1.76	14.93 ± 1.88	14.81 ± 2.06
	MASD (mm)	2.22 ± 0.52	2.34 ± 0.59	2.38 ± 0.71

FET1CASE2				
	DSC	0.72 ± 0.08	0.87 ± 0.04	0.88 ± 0.04
	JAC	0.57 ± 0.09	0.77 ± 0.07	0.78 ± 0.06
	OV	0.95 ± 0.09	0.98 ± 0.02	0.99 ± 0.02
	HD (mm)	13.26 ± 3.25	12.25 ± 3.29	12.39 ± 3.19
	MASD (mm)	3.09 ± 0.84	2.57 ± 0.89	2.66 ± 0.88

FET1CASE3				
	DSC	0.66 ± 0.06	0.82 ± 0.05	0.84 ± 0.04
	JAC	0.50 ± 0.08	0.69 ± 0.07	0.72 ± 0.06
	OV	0.99 ± 0.01	0.97 ± 0.04	0.98 ± 0.04
	HD (mm)	8.83 ± 1.12	9.09 ± 2.09	9.33 ± 2.46
	MASD (mm)	2.86 ± 0.63	2.49 ± 0.75	2.68 ± 0.73

ALL				
	DSC	0.71 ± 0.07	0.85 ± 0.05	0.87 ± 0.04
	JAC	0.55 ± 0.09	0.75 ± 0.07	0.77 ± 0.07
	OV	0.97 ± 0.07	0.97 ± 0.03	0.98 ± 0.02
	HD (mm)	12.75 ± 3.54	12.26 ± 3.44	12.33 ± 3.42
	MASD (mm)	2.72 ± 0.78	2.47 ± 0.76	2.57 ± 0.79

DSC Dice Similarity Coefficient, JAC Jaccard Index, OV Overlap Volume, HD Hausdorff Distance, MASD Mean Absolute Surface Distance.

### 3.5 Radiotherapy plan analysis

Analysis of the ten standard-of-care radiotherapy plans revealed median D_2%_, D_50%_ and D_98%_ of the PTV as 62.50 (61.46–63.33) Gy, 60.75 (60.13–61.70) Gy, and 58.13 (56.77–59.18) Gy, respectively. The median V_95%_, conformity index, and homogeneity index was 322.95 (318.8–324.5) cc, 0.99 (0.98–1.00) and 7.61 (4.40–10.14), respectively. Dose volume histograms for each centre’s PTV, optic chiasm, and brainstem can be found in Supplementary Fig. 6. Information on other OARs can be found in Supplementary Table 13.

## 4 Discussion

The incorporation of FET PET with standard-of-care MRI for newly diagnosed glioblastoma adjuvant RT planning may substantially inform both GTV and CTV derivation through identifying disease otherwise occult on T1c. This could lead to potential improvements in both tumour local control and sparing of healthy brain tissue. To our knowledge, the FIG trial represents the largest prospective multi-centre study in newly diagnosed adults with glioblastoma. In the FIG study, participants receive RT as per standard-of-care, with TV_MR+FET_ delineation performed post-RT. This will be a two-step process requiring BTV interpretation and delineation by a credentialed site NMP followed by sequential incorporation of said BTV by the credentialed site RO to create TV_MR+FET_. The FIG trial’s nuclear medicine credentialing identified and addressed potential sources of error in BTV delineation by participating NMPs [19]. Similarly, the feasibility of TV_MR+FET_ delineation had to be assessed**.** Therefore, the creation of the BTV for each of the three cases was fixed, utilising a sole NMP expert, attributing any subsequent variability to the ROs alone.

After central review and resubmission where indicated, all ROs and sites successfully passed the credentialing components, demonstrating the feasibility of TV_MR+FET_ delineation. Further quantitative assessment showed TV_MR+FET_ to be significantly larger than TV_MR_, with greater volume, spatial and boundary agreement for GTV_MR+FET_ compared to GTV_MR_. As the NMP-derived BTV was identical for each case for incorporation by ROs, higher agreement may have been expected for GTV_MR+FET_. This was not observed, however, for FET1CASE3, noting decreased boundary agreement primarily contributed by an outlier contour. Additionally, importing and registration of PET data to CT/MRI within each site’s TPS and conjoining MR-derived GTV with the supplied BTV may have constituted minor sources of variability. Furthermore, MASD was higher for CTV_MR+FET_, possibly attributed to RO preferences in margin size, as the instructed GTV_MR+FET_-to-CTV_MR+FET_ expansion was 10–15 mm. The TV_paired_ analysis highlighted individual RO spatial overlap and boundary differences between respectively derived TV_MR_ and TV_MR+FET_ contours. Pleasingly, the average OV for GTV_paired_ was close to one (OV = 0.97), demonstrating that ROs could reproducibly incorporate the tumour bed plus residual enhancement into GTV_MR+FET_. Low agreement in delineation of the optic chiasm was noted as part of the credentialing program, reflecting in part, the small size of this structure [29], [30]. Given the chiasm is a critical organ at risk, the importance of its accurate delineation has been reinforced during the prospective phase of the study.

Dissaux *et al*. (2022) separately assessed FET PET and multiparametric MRI for TV delineation in 30 patients with newly diagnosed GBM. Three NMPs and three radiologists respectively reported a mean DSC of 0.841 and 0.922 for T1c and FET PET [31]. However, inter-observer assessment of combined (MR+FET) target delineation was not assessed. In the present study, a mean of 0.83 was found for T1c, increasing to 0.85 when incorporating both MRI and FET PET. As stated previously, the spatial overlap of GTV_MR+FET_ was expected to be higher than GTV_MR_; however, given the ROs involved had zero/minimal prior experience with FET PET data (84.2 %), this may have reduced agreement.

The impact of FET PET on adjuvant RT planning has been investigated in a series of studies. Niyazi *et al*. (2011) reported consistently larger FET PET-derived BTVs compared to MRI-derived GTVs [16]. Furthermore, JAC (or intersection over union) of MRI-derived CTV and FET PET-derived CTV (BTV + 20 mm) was significantly different from unity, and combined MRI+FET CTV was larger compared to MRI-derived CTV. Harat *et al.* (2016) further found FET PET-derived BTV to be correspondingly larger than MRI-derived GTV [13]. Spatial overlap between these volumes also showed poor concordance prior to treatment, and at baseline and recurrence [32], [33].

Concordance with post-RT sites of progressive disease are more likely to be encompassed by FET PET volumes compared to MRI T1c, as FET PET offers an alternative option to visualising tumour physiology [34]. Niyazi *et al.* (2012) assessed the combined use of FET PET and MRI for detection of tumour recurrence with 49.4 % of recurrences found to be in-field, 12.6 % out-of-field, and 3.8 % marginal (34.2 % no relapse during follow up) [16]. Lundemann *et al*. (2017) analysed patterns of recurrence as 82 % central, 10 % in-field, 2 % marginal and 6 % distant. Expansion of the combined MRI and FET PET-derived GTV of 12 mm would reclassify recurrences as central in 82 % of patients [17]. The accuracy of correlating pre- and post-treatment volumes may be somewhat limited by post-operative anatomical changes in the resection cavity. Furthermore, the pattern and timing of recurrences is influenced by isocitrate dehydrogenase mutation status and O(6)-methylguanine-DNA methyltransferase methylation, with methylated patients often having longer progression free survival and exhibiting more remote recurrences [16], [35], [36].

FET PET may inform RT margin optimisation allowing for reduced dose to the normal brain tissue with potentially comparable treatment outcomes. Allard *et al*. (2023) reported post-operative FET PET to have better spatial overlap with MRI-determined areas of progressive tumour in patients with biopsy/partial resection compared to those with total/subtotal resection [37]. Fleischmann *et al*. (2020) found that the minimal margin to encompass recurrent contrast enhancing tumour was less for MR+FET-derived GTVs compared to MR-derived GTVs [38]. For FET PET-guided boost irradiation, Piroth et al. (2016) reported that FET PET at radiation treatment planning (plus 7 mm margin) showed better consistency encapsulating recurrent FET PET defined tumour compared to baseline T1c MRI with the same margin [33]. FET PET information may result in a volumetrically larger GTV; however, CTV optimisation may be equal to, or even smaller than, MR-derived CTVs depending on the CTV margin applied.

This study was limited to three credentialing cases, although these cases were chosen to reflect diverse clinical scenarios. That said, there were 19 ROs across 10 study sites taking part, representing a significant undertaking. We acknowledge that the credentialing program did permit a differential, tighter margin (10–15 mm) in the hybrid volumes compared to standard-of-care [4], [23]. However, despite this, the hybrid volumes were consistently volumetrically larger than standard TVs. Importantly, T2/FLAIR changes on MRI were included in the CTV to account for microscopic disease; however, FET PET highlighting non-enhancing tumour may be another cause for observer discrepancies [39]. Using multiple, central NMP to contour the BTV would have better reflected the real-world setting across multiple sites, however this would have introduced an additional source of variability and confounded the analysis of variability amongst the RO cohort.

All participating FIG study sites successfully passed credentialing requirements, resulting in increased familiarity with FET PET imaging and experience with incorporating NMP-derived BTV into adjuvant TVs for glioblastoma. The resulting central review and resubmission process has shown this collaborative delineation approach to be feasible. Important learnings from the RO credentialing program have been incorporated into the prospective phase of the FIG study.

## Funding

The FIG study is supported by the Medical Research Future Fund (MRFF) (Grant No. MRF1152501), MRFF Australian Brain Cancer Mission: Innovative Trials Grant MRF9500003, Cure Brain Cancer Foundation, the Victorian Cancer Agency Centre for Research Excellence in Brain Cancer, Cyclotek and Telix Pharmaceuticals. NB gratefully acknowledges the award of the RTP scholarship from the University of Western Australia. NB is supported by a Cancer Council WA PhD Top Up Scholarship. AMS is supported by NHMRC Investigator Grant No 1177837. Special thanks to Anita and Anthony Parise for their generous contribution to brain cancer research at Sir Charles Gairdner Hospital.

## CRediT authorship contribution statement

**Nathaniel Barry:** Methodology, Formal analysis, Investigation, Writing – original draft, Writing – review & editing, Visualization. **Eng-Siew Koh:** Conceptualization, Methodology, Investigation, Writing – review & editing, Supervision, Project administration. **Martin A. Ebert:** Conceptualization, Methodology, Writing – review & editing. **Alisha Moore:** Investigation, Data curation, Methodology, Writing – review & editing. **Roslyn J. Francis:** Conceptualization, Methodology, Investigation, Writing – review & editing. **Pejman Rowshanfarzad:** Writing – review & editing. **Ghulam Mubashar Hassan:** Writing – review & editing. **Sweet P. Ng:** Investigation, Writing – review & editing. **Michael Back:** Investigation. **Benjamin Chua:** Investigation, Writing – review & editing. **Mark B. Pinkham:** Investigation, Writing – review & editing. **Andrew Pullar:** Investigation, Writing – review & editing. **Claire Phillips:** Investigation, Writing – review & editing. **Joseph Sia:** Investigation, Writing – review & editing. **Peter Gorayski:** Investigation, Writing – review & editing. **Hien Le:** Investigation, Writing – review & editing. **Suki Gill:** Investigation, Writing – review & editing. **Jeremy Croker:** Investigation, Writing – review & editing. **Nicholas Bucknell:** Investigation, Writing – review & editing. **Catherine Bettington:** Investigation, Writing – review & editing. **Farhan Syed:** Investigation, Writing – review & editing. **Kylie Jung:** Investigation, Writing – review & editing. **Joe Chang:** Investigation, Writing – review & editing. **Andrej Bece:** Investigation. **Catherine Clark:** Investigation, Writing – review & editing. **Mori Wada:** Investigation, Writing – review & editing. **Olivia Cook:** Investigation, Data curation. **Angela Whitehead:** Investigation, Data curation. **Alana Rossi:** Investigation, Data curation. **Andrew Grose:** Investigation, Data curation. **Andrew M. Scott:** Conceptualization, Methodology, Writing – review & editing, Supervision, Project administration, Funding acquisition.

## Declaration of Competing Interest

The authors declare that they have no known competing financial interests or personal relationships that could have appeared to influence the work reported in this paper.

## Footnotes

1 https://plastimatch.org/ (version 1.9.0).

2 https://github.com/pyplati/platipy (version 0.4.1).

3 https://rpy2.github.io/ (version 3.5.4).
