# Supplementary material for: [18]F-fluoroethyl-l-tyrosine positron emission tomography for radiotherapy target delineation: Results from a Radiation Oncology credentialing program
Source: Phys Imaging Radiat Oncol. 2024 Mar 13;30:100568. doi: 10.1016/j.phro.2024.100568 (PMC10998205; doi:10.1016/j.phro.2024.100568)
Supplement: Supplementary data 1 [file mmc1.docx]

Let A and B represent a set of voxels from each respective segmentation:

$$\boldsymbol{DSC}\left( \boldsymbol{A,B} \right)\boldsymbol{=}\frac{\boldsymbol{2(A\cap B)}}{\boldsymbol{A+B}}$$

$$\boldsymbol{JAC}\left( \boldsymbol{A,B} \right)\boldsymbol{=}\frac{\boldsymbol{(A\cap B)}}{\boldsymbol{(A\cup B)}}$$

$$\boldsymbol{OV}\left( \boldsymbol{A,B} \right)\boldsymbol{=}\frac{\boldsymbol{(A\cap B)}}{\boldsymbol{min(A,B)}}$$

Let S(Y) denote the set of surface voxels of Y. The shortest distance of a voxel x to S(Y) is defined as:

$$\boldsymbol{d}\left( \boldsymbol{x,S}\left( \boldsymbol{Y} \right) \right)\boldsymbol{=}\min_{\boldsymbol{y}\in S(Y)} \left\| \boldsymbol{x-y} \right\|$$

Where $\left\| . \right\|$ denotes Euclidean distance. Then Hausdorff distance (d_H_) and mean absolute surface distance (MASD) are defined as:

$$\boldsymbol{d}_{\boldsymbol{H}}\left( \boldsymbol{A,B} \right)\boldsymbol{=max}\left\{ \max_{\boldsymbol{a}\in S(A)} \boldsymbol{d}\left( \boldsymbol{a,S(B)} \right)\boldsymbol{,}\max_{\boldsymbol{b}\in S(B)} \boldsymbol{d}\left( \boldsymbol{b, S(A)} \right) \right\}$$

$$\boldsymbol{MASD}\left( \boldsymbol{A,B} \right)\boldsymbol{=}\frac{\boldsymbol{1}}{\boldsymbol{A+B}}\left\{ \sum_{\boldsymbol{a}\in S(A)} \boldsymbol{d(a,S(B))}\boldsymbol{+}\sum_{\boldsymbol{b}\in S(B)} \boldsymbol{d(b,S(A))} \right\}$$

From McGraw and Wong:

$$\boldsymbol{ICC}\left( \boldsymbol{2,1} \right)\boldsymbol{=}\frac{\boldsymbol{M}\boldsymbol{S}_{\boldsymbol{R}}\boldsymbol{-M}\boldsymbol{S}_{\boldsymbol{E}}}{\boldsymbol{M}\boldsymbol{S}_{\boldsymbol{R}}\boldsymbol{+}\left( \boldsymbol{k-1} \right)\boldsymbol{M}\boldsymbol{S}_{\boldsymbol{E}}\boldsymbol{+}\frac{\boldsymbol{k}}{\boldsymbol{n}}\boldsymbol{(M}\boldsymbol{S}_{\boldsymbol{C}}\boldsymbol{-M}\boldsymbol{S}_{\boldsymbol{E}}\boldsymbol{)}}$$

Where MS_R_ is the mean square for rows (between subjects), MS_E_ is the mean square for error, MS_C_ is the mean square for columns (between raters/measurements), n is the number of subjects, and k is the number of raters/measurements. The ICC ranges from 0 to 1, with 1 being perfect reliability.

Radiotherapy metrics definitions:

$$\boldsymbol{D}_{\boldsymbol{x\%}}$$

Dose to x percent of the PTV.

$$\boldsymbol{V}_{\boldsymbol{x\%}}$$

Volume that received x percent of the prescribed dose.

The conformity index (CI) is defined as:

$$\boldsymbol{CI=}\frac{\boldsymbol{V}_{\boldsymbol{95\%}}}{\boldsymbol{PTV}}$$

Where PTV is the planning target volume.

The homogeneity index (HI) is defined as:

$$\boldsymbol{HI=}\frac{\boldsymbol{D}_{\boldsymbol{2\%}}\boldsymbol{-}\boldsymbol{D}_{\boldsymbol{98\%}}}{\boldsymbol{Prescribed Dose (Gy)}}$$

**Supplementary Table 1.** MRI acquisition parameters.

|  | **Machine** | **Sequence** | **Slice thickness (mm)** | **TR (ms)** | **TE (ms)** | **TI (ms)** | **Matrix** |
| --- | --- | --- | --- | --- | --- | --- | --- |
| FET1CASE1 | 3T Philips Ingenia | T1c MPRAGE | 1 | 6.132 | 2.781 |  | 256 x 256 x 180 |
|  |  | T2 Axial TSE | 4 | 4718 | 120 |  | 768 x 768 x 28 |
| FET1CASE2 | 1.5T Siemens Aera | T1c Axial TSE | 3 | 1050 | 10 |  | 512 x 512 x 60 |
|  |  | T2 Axial FLAIR MPR | 2 | 5000 | 335 | 1800 | 256 x 256 x 79 |
| FET1CASE3 | 1.5T Siemens Aera | T1c MPRAGE | 1 | 1910 | 3.14 | 1100 | 256 x 256 x 176 |
|  |  | T2 Axial FLAIR MPR | 2 | 5000 | 335 | 1800 | 256 x 256 x 75 |

**Supplementary Table 2.** Organs at risk (OARs) contouring guidelines outlined by the radiotherapy and quality assurance (RTQA) manual.

| **OAR** | **Standardised Name Contouring Guideline** | |
| --- | --- | --- |
| **Brainstem** | Brainstem | The brainstem comprises the midbrain, pons and medulla oblongata. It starts from the superior limit of the posterior clinoids to the inferior limit of the foramen magnum. It continues superiorly with the diencephalon and inferiorly with the spinal cord |
| **Optic Chiasm** | OpticChiasm | The convergence of the optic nerves in front and the divergence of the optic tracts behind. With conventional CT or MRI, the optic tracts are visible for only 1–2 cm posterior to the optic chiasm before the fibres spread and blend into the rest of the brain parenchyma. |
| **Lens** | Lens_L Lens_R | The lens of the eye is a biconvex avascular structure, located between the vitreous and the iris. Its diameter measures up to 10 mm. |
| **Eye** | Eye_L Eye_R | Each eye should be contoured separately. Contour the entire eye, to include the anterior and posterior segments. Anteriorly, the structure consists of the cornea, iris, ciliary body, and lens. Posteriorly, the eye includes the anterior hyaloid membrane, vitreous humor, retina, and choroid. Exclude the optic nerve and extra-ocular muscles. |
| **Retina** | Retina_L Retina_R | It is an approximately 0.25 mm-thick neurosensorial membrane with a rich vascularisation and it lines the posterior wall of the eye. The retina cannot be directly visualised on the standard MRI sequences for the orbit. It can be drawn as a membrane that lays in the posterior 5/6 of the bulb, extending nearly as far as the ciliary body. On axial images the anterior limit of the retina is between the insertion of the medial rectus muscle and the insertion of the lateral rectus muscle, posteriorly to the ciliary body |
| **Optic Nerve** | OpticNerve_L OpticNerve_R | The optic nerve is usually 2–5 mm thick and in general is clearly identifiable on CT. It must be contoured all the way from the posterior edge of the eyeball, through the bony optic canal to the optic chiasm. MRI is recommended for a better delineation of the optic nerve, at least close to the optic chiasm. |

**Supplementary Table 3.** Standard-of-care radiation therapy target volume definitions as specified by the FIG trial RTQA manual.

| **Structure Name** | **Standardised Name** | **Definition** |
| --- | --- | --- |
| Gross Tumour Volume | GTV | The GTV is the volume encompassed by the surgical cavity and any enhancing tissue as defined on the post-operative T1 gadolinium-enhanced MRI sequence. T2 FLAIR changes/abnormalities thought to represent tumour should also be incorporated.  In case of complete or subtotal removal, the position of the tumour bed can have shifted, and the GTV should take the new position of the abnormalities on the planning CT scan and any post-operative imaging into account. The pre and post-operative MRI imaging (within 0-72 hours), as well as the planning CT scan, should aid in the definition of the GTV. If the T2/FLAIR MRI changes are thought to represent macroscopic tumour, then these should be incorporated at the discretion of the treating RO. |
| GTV 60Gy/30fx prescription* | GTV6000 |  |
| GTV 40.05Gy/15fx prescription* | GTV4005 |  |
| MRI-Derived GTV* | GTV_MR | Utilized for radiotherapy treatment and delineated by RO. |
| Clinical Target Volume* | CTV | The Clinical Target Volume (CTV) is defined by a 1.5cm volumetric expansion of the GTV. The CTV extends to the contralateral hemisphere only when midline structures such as the corpus callosum and the contralateral hemisphere are invaded by tumour. The tentorium and meninges should be considered as anatomical borders and therefore a margin of 0-0.5cm is sufficient to encompass the microscopic spread at these borders. Volumetric expansion may also be reduced in areas adjacent to sensitive structures.  Oedema seen on T2-weighted MRI (FLAIR sequence) may be considered when delineating the CTV volume at the discretion of the treating RO. |
| CTV 60Gy/30fx prescription* | CTV6000 |  |
| CTV 40.05Gy/15fx prescription* | CTV4005 |  |
| Planning Target Volume* | PTV | The Planning Target Volume (PTV) will take into account uncertainties of planning and setup. This margin should be based upon known departmental values, but will usually be of the order of 0.3 cm. All margins should be added using a three-dimensional (3-D) growth algorithm where possible. |
| PTV 60Gy/30fx prescription* | PTV6000 |  |
| PTV 40.05Gy/15fx prescription* | PTV4005 |  |

**Patients with multifocal disease should have a single composite GTV, CTV and PTV contoured.*

**Supplementary Table 4.** Hybrid radiation therapy target volume definitions as specified by the FIG trial RTQA manual.

| **Research Structure** | **Standardised Name+** | **Definition** |
| --- | --- | --- |
| FET-PET Derived GTV - delineated by nuclear medicine physician post-treatment  *NOT used for radiotherapy treatment | Static_GTV_Final | FET-PET positive volume*  **The NM physician must use MIM v7.0 (or higher version updates) and the FIG MIM Workflow to complete this activity Volume must be exported and uploaded to TROG QA for Central Review/approval prior to RO volume delineation* |
| FET-PET and MR derived GTV - delineated by radiation oncologist post-treatment with access to Static_GTV_Final  *NOT used for radiotherapy treatment | RO_GTVXXXX_PT1MR1 | Using the Static_GTV_Final, generate a new structure, RO_GTV_PT1MR1* **The treating clinician should make any adjustments necessary (according to adjacent critical structures etc.) and imaging available, noting the Static_GTV_Final may not be a contiguous closed volume.* |
| FET-PET and MR derived CTV - delineated by radiation oncologist post-treatment (may be identical to RO_GTVXXXX_PT1MR1)  *NOT used for radiotherapy treatment | RO_CTVXXXX_PT1MR1 | (RO_GTVXXXX_PT1MR1) + 1-1.5cm margin |
| FET-PET and MR derived PTV - delineated by radiation oncologist post-treatment  *NOT used for radiotherapy treatment | RO_PTVXXXX_PT1MR1 | (RO_CTVXXXX_PT1MR1) + 0.3cm margin |

**Supplementary Table 5.** Radiation schedule 60 Gy / 30 daily fractions dose goals for target volumes and dose constraints for organs at risk (OARs):

| **Target** | **Parameter** | **Goal** |
| --- | --- | --- |
| **GTV** | D98% | ≥ 58.8Gy |
|  | D99% | ≥ 57Gy |
|  | D2% | ≤ 64.2Gy |
| **PTV** | D98% | ≥ 57Gy |
|  | D99% | ≥ 55.8Gy |
|  | D2% | ≤ 64.2Gy |
| **OARs** | **Parameter** | **Goal** |
| **Brainstem** | D0.03cc | ≤ 56Gy |
| **Left Eye; Right Eye** | D0.03cc | ≤ 45Gy |
| **Left Lens; Right Lens** | D0.03cc | ≤ 6Gy |
| **Left Retina; Right Retina** | D0.03cc | ≤ 36Gy |
| **Left Optic Nerve; Right Optic Nerve** | D0.03cc | ≤ 56Gy |
| **Optic Chiasm** | D0.03cc | ≤ 56Gy |

**Supplementary Table 6.** Treatment planning system software information utilised for the radiation oncology quality assurance program.

| **Treatment Planning System** | **Number** | **Version(s)** |
| --- | --- | --- |
| Monaco, Elekta (Stockholm, Sweden) | 1 | V5.11.03 |
| Eclipse, Varian (Palo Alto, California) | 6 | V15.6, V16.01.04, V13.7.29, V16.1.4, V15.6.03, V15.6.8 |
| Pinnacle, Philips (Amsterdam, Netherlands) | 2 | V16.2.1 (both) |
| RayStation, RaySearch Laboratories (Stockholm, Sweden) | 1 | V8.1.2.5 |

**Supplementary Table 7.** Distribution of volumes from required contours as part of RO credentialing for all three cases.

| Structure | FET1CASE1 | FET1CASE2 | FET1CASE3 | ALL |
| --- | --- | --- | --- | --- |
| Brainstem | 28.31 +/- 2.7 | 28.54 +/- 4.69 | 28.15 +/- 2.71 | 28.35 +/- 3.55 |
| Brainstem | 28.41 (22.39 - 32.78) | 28.24 (15.81 - 35.33) | 27.79 (24.41 - 33.39) | 28.16 (15.81 - 35.33) |
| OpticChiasm | 0.64 +/- 0.28 | 1.03 +/- 0.55 | 0.76 +/- 0.32 | 0.81 +/- 0.44 |
| OpticChiasm | 0.58 (0.17 - 1.39) | 0.92 (0.22 - 2.38) | 0.77 (0.25 - 1.59) | 0.77 (0.17 - 2.38) |
| OpticNerve_L | 0.68 +/- 0.3 | None | None | 0.68 +/- 0.3 |
| OpticNerve_L | 0.64 (0.16 - 1.61) | None | None | 0.64 (0.16 - 1.61) |
| OpticNerve_R | 0.6 +/- 0.19 | None | None | 0.60 +/- 0.19 |
| OpticNerve_R | 0.59 (0.28 - 0.93) | None | None | 0.59 (0.28 - 0.93) |
| Retina_L | 2.4 +/- 0.99 | None | None | 2.40 +/- 0.99 |
| Retina_L | 2.36 (0.94 - 4.67) | None | None | 2.36 (0.94 - 4.67) |
| Retina_R | 2.42 +/- 1.09 | None | None | 2.42 +/- 1.09 |
| Retina_R | 2.57 (0.94 - 4.59) | None | None | 2.57 (0.94 - 4.59) |
| Eye_L | 8.56 +/- 0.82 | None | None | 8.56 +/- 0.82 |
| Eye_L | 8.73 (6.98 - 9.85) | None | None | 8.73 (6.98 - 9.85) |
| Eye_R | 8.87 +/- 0.67 | None | None | 8.87 +/- 0.67 |
| Eye_R | 8.95 (7.49 - 9.99) | None | None | 8.95 (7.49 - 9.99) |
| Lens_L | 0.24 +/- 0.14 | None | None | 0.24 +/- 0.14 |
| Lens_L | 0.22 (0.04 - 0.59) | None | None | 0.22 (0.04 - 0.59) |
| Lens_R | 0.22 +/- 0.15 | None | None | 0.22 +/- 0.15 |
| Lens_R | 0.18 (0.04 - 0.6) | None | None | 0.18 (0.04 - 0.6) |
| CLINICAL_GTV | 23.4 +/- 4.4 | 51.09 +/- 10.04 | 8.11 +/- 0.91 | 28.61 +/- 18.82 |
| CLINICAL_GTV | 22.83 (16.92 - 38.66) | 50.42 (39.89 - 82.69) | 8.0 (7.1 - 11.07) | 23.22 (7.1 - 82.69) |
| HYBRID_GTV | 38.43 +/- 4.06 | 82.46 +/- 10.23 | 16.43 +/- 1.97 | 47.40 +/- 28.08 |
| HYBRID_GTV | 38.52 (30.96 - 47.84) | 80.75 (62.15 - 103.33) | 16.8 (9.97 - 19.14) | 39.31 (9.97 - 103.33) |
| CLINICAL_CTV | 163.03 +/- 14.49 | 203.24 +/- 31.15 | 58.61 +/- 8.02 | 146.24 +/- 62.86 |
| CLINICAL_CTV | 158.44 (144.07 - 197.72) | 197.86 (169.17 - 301.67) | 56.41 (45.56 - 80.9) | 163.08 (45.56 - 301.67) |
| HYBRID_CTV | 194.98 +/- 17.67 | 256.32 +/- 28.46 | 80.23 +/- 11.51 | 182.56 +/- 74.2 |
| HYBRID_CTV | 190.71 (144.76 - 221.37) | 252.28 (195.12 - 314.59) | 79.93 (54.44 - 99.05) | 196.44 (54.44 - 314.59) |
| CLINICAL_PTV | 219.91 +/- 19.4 | 268.98 +/- 41.61 | 86.59 +/- 10.43 | 197.67 +/- 79.96 |
| CLINICAL_PTV | 210.84 (198.33 - 265.65) | 260.25 (223.77 - 390.57) | 85.03 (68.47 - 115.77) | 219.66 (68.47 - 390.57) |
| HYBRID_PTV | 264.18 +/- 24.49 | 333.55 +/- 41.3 | 115.39 +/- 16.37 | 244.5 +/- 93.57 |
| HYBRID_PTV | 258.96 (202.14 - 302.4) | 326.39 (258.44 - 433.51) | 115.32 (80.84 - 145.17) | 262.05 (80.84 - 433.51) |

**N.B.** In the following tables ***PAIRED*** means the respective result from assessing spatial overlap and boundary agreement for each observers’ pair of standard/clinical target volumes and their hybrid target volumes. This does not apply to Table e7.

**Supplementary Table 8.** Results of pairwise calculation of the Dice Similarity Coefficient (DSC) to assess spatial overlap of contours delineated for each of the three credentialing cases.

| Structure | FET1CASE1 | FET1CASE2 | FET1CASE3 | ALL |
| --- | --- | --- | --- | --- |
| Brainstem | 0.87 +/- 0.04 | 0.81 +/- 0.07 | 0.87 +/- 0.03 | 0.85 +/- 0.06 |
| Brainstem | 0.87 (0.75 - 0.99) | 0.83 (0.59 - 0.99) | 0.86 (0.81 - 0.94) | 0.86 (0.59 - 0.99) |
| OpticChiasm | 0.46 +/- 0.25 | 0.42 +/- 0.21 | 0.28 +/- 0.26 | 0.41 +/- 0.24 |
| OpticChiasm | 0.51 (0.0 - 0.84) | 0.46 (0.0 - 0.85) | 0.24 (0.0 - 0.77) | 0.46 (0.0 - 0.85) |
| OpticNerve_L | 0.56 +/- 0.13 | None | None | 0.56 +/- 0.13 |
| OpticNerve_L | 0.58 (0.15 - 0.8) | None | None | 0.58 (0.15 - 0.8) |
| OpticNerve_R | 0.58 +/- 0.13 | None | None | 0.58 +/- 0.13 |
| OpticNerve_R | 0.59 (0.24 - 0.99) | None | None | 0.59 (0.24 - 0.99) |
| Retina_L | 0.48 +/- 0.14 | None | None | 0.48 +/- 0.14 |
| Retina_L | 0.47 (0.09 - 0.99) | None | None | 0.47 (0.09 - 0.99) |
| Retina_R | 0.47 +/- 0.16 | None | None | 0.47 +/- 0.16 |
| Retina_R | 0.48 (0.11 - 0.99) | None | None | 0.48 (0.11 - 0.99) |
| Eye_L | 0.9 +/- 0.03 | None | None | 0.90 +/- 0.03 |
| Eye_L | 0.9 (0.82 - 0.99) | None | None | 0.90 (0.82 - 0.99) |
| Eye_R | 0.91 +/- 0.03 | None | None | 0.91 +/- 0.03 |
| Eye_R | 0.92 (0.86 - 0.99) | None | None | 0.92 (0.86 - 0.99) |
| Lens_L | 0.66 +/- 0.17 | None | None | 0.66 +/- 0.17 |
| Lens_L | 0.68 (0.19 - 1.0) | None | None | 0.68 (0.19 - 1.0) |
| Lens_R | 0.66 +/- 0.19 | None | None | 0.66 +/- 0.19 |
| Lens_R | 0.69 (0.18 - 1.0) | None | None | 0.69 (0.18 - 1.0) |
| CLINICAL_GTV | 0.81 +/- 0.06 | 0.84 +/- 0.06 | 0.87 +/- 0.06 | 0.83 +/- 0.06 |
| CLINICAL_GTV | 0.83 (0.6 - 0.91) | 0.85 (0.64 - 0.93) | 0.87 (0.69 - 0.96) | 0.85 (0.6 - 0.96) |
| HYBRID_GTV | 0.84 +/- 0.04 | 0.86 +/- 0.06 | 0.86 +/- 0.07 | 0.85 +/- 0.05 |
| HYBRID_GTV | 0.84 (0.71 - 0.95) | 0.86 (0.69 - 0.96) | 0.87 (0.66 - 0.98) | 0.86 (0.66 - 0.98) |
| CLINICAL_CTV | 0.92 +/- 0.04 | 0.89 +/- 0.05 | 0.88 +/- 0.06 | 0.90 +/- 0.05 |
| CLINICAL_CTV | 0.93 (0.78 - 0.97) | 0.91 (0.71 - 0.97) | 0.89 (0.72 - 0.98) | 0.91 (0.71 - 0.98) |
| HYBRID_CTV | 0.91 +/- 0.04 | 0.9 +/- 0.04 | 0.87 +/- 0.07 | 0.89 +/- 0.05 |
| HYBRID_CTV | 0.92 (0.76 - 0.96) | 0.9 (0.76 - 0.98) | 0.89 (0.69 - 0.98) | 0.91 (0.69 - 0.98) |
| CLINICAL_PTV | 0.93 +/- 0.03 | 0.89 +/- 0.05 | 0.89 +/- 0.05 | 0.90 +/- 0.05 |
| CLINICAL_PTV | 0.93 (0.85 - 0.97) | 0.91 (0.73 - 0.97) | 0.89 (0.75 - 0.98) | 0.92 (0.73 - 0.98) |
| HYBRID_PTV | 0.91 +/- 0.04 | 0.9 +/- 0.04 | 0.88 +/- 0.06 | 0.90 +/- 0.05 |
| HYBRID_PTV | 0.92 (0.77 - 0.97) | 0.9 (0.75 - 0.98) | 0.9 (0.72 - 0.99) | 0.91 (0.72 - 0.99) |
| PAIRED_GTV | 0.72 +/- 0.06 | 0.72 +/- 0.08 | 0.66 +/- 0.06 | 0.71 +/- 0.07 |
| PAIRED_GTV | 0.71 (0.61 - 0.89) | 0.74 (0.51 - 0.89) | 0.66 (0.59 - 0.85) | 0.70 (0.51 - 0.89) |
| PAIRED_CTV | 0.87 +/- 0.03 | 0.87 +/- 0.04 | 0.82 +/- 0.05 | 0.85 +/- 0.05 |
| PAIRED_CTV | 0.87 (0.79 - 0.92) | 0.86 (0.78 - 0.95) | 0.81 (0.74 - 0.92) | 0.85 (0.74 - 0.95) |
| PAIRED_PTV | 0.89 +/- 0.03 | 0.88 +/- 0.04 | 0.84 +/- 0.04 | 0.87 +/- 0.04 |
| PAIRED_PTV | 0.89 (0.8 - 0.96) | 0.87 (0.79 - 0.94) | 0.83 (0.77 - 0.93) | 0.87 (0.77 - 0.96) |


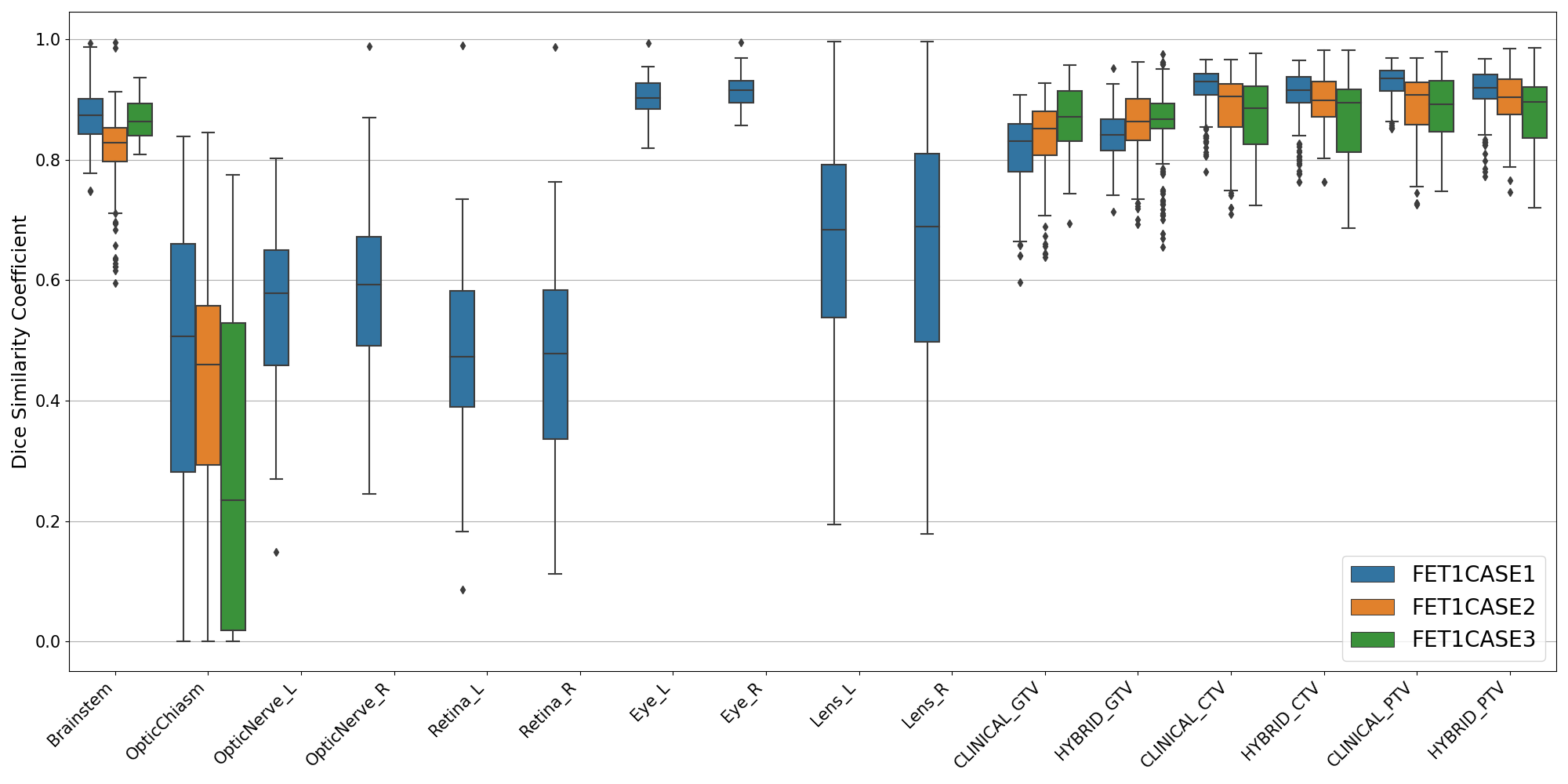


**Supplementary Figure 1.** Boxplots visualising the distribution of pairwise Dice Similarity Coefficient (DSC) scores for each of the required contours, grouped by the three credentialing cases.

**Supplementary Table 9.**  Results of pairwise calculation of Jaccard Index (JAC) to assess spatial overlap of contours delineated for each of the three credentialing cases.

| Structure | FET1CASE1 | FET1CASE2 | FET1CASE3 | ALL |
| --- | --- | --- | --- | --- |
| Brainstem | 0.77 +/- 0.06 | 0.69 +/- 0.09 | 0.77 +/- 0.05 | 0.74 +/- 0.08 |
| Brainstem | 0.78 (0.6 - 0.99) | 0.71 (0.42 - 0.99) | 0.76 (0.68 - 0.88) | 0.75 (0.42 - 0.99) |
| OpticChiasm | 0.33 +/- 0.2 | 0.29 +/- 0.16 | 0.19 +/- 0.19 | 0.29 +/- 0.19 |
| OpticChiasm | 0.34 (0.0 - 0.72) | 0.3 (0.0 - 0.73) | 0.13 (0.0 - 0.63) | 0.3 (0.0 - 0.73) |
| OpticNerve_L | 0.4 +/- 0.13 | None | None | 0.4 +/- 0.13 |
| OpticNerve_L | 0.41 (0.08 - 0.67) | None | None | 0.41 (0.08 - 0.67) |
| OpticNerve_R | 0.43 +/- 0.14 | None | None | 0.43 +/- 0.14 |
| OpticNerve_R | 0.42 (0.14 - 0.98) | None | None | 0.42 (0.14 - 0.98) |
| Retina_L | 0.32 +/- 0.12 | None | None | 0.32 +/- 0.12 |
| Retina_L | 0.31 (0.05 - 0.98) | None | None | 0.31 (0.05 - 0.98) |
| Retina_R | 0.32 +/- 0.14 | None | None | 0.32 +/- 0.14 |
| Retina_R | 0.31 (0.06 - 0.98) | None | None | 0.31 (0.06 - 0.98) |
| Eye_L | 0.83 +/- 0.05 | None | None | 0.83 +/- 0.05 |
| Eye_L | 0.82 (0.69 - 0.99) | None | None | 0.82 (0.69 - 0.99) |
| Eye_R | 0.84 +/- 0.04 | None | None | 0.84 +/- 0.04 |
| Eye_R | 0.84 (0.75 - 0.99) | None | None | 0.84 (0.75 - 0.99) |
| Lens_L | 0.51 +/- 0.18 | None | None | 0.51 +/- 0.18 |
| Lens_L | 0.52 (0.11 - 0.99) | None | None | 0.52 (0.11 - 0.99) |
| Lens_R | 0.52 +/- 0.2 | None | None | 0.52 +/- 0.2 |
| Lens_R | 0.53 (0.1 - 0.99) | None | None | 0.53 (0.1 - 0.99) |
| CLINICAL_GTV | 0.69 +/- 0.09 | 0.72 +/- 0.08 | 0.77 +/- 0.09 | 0.72 +/- 0.09 |
| CLINICAL_GTV | 0.71 (0.43 - 0.83) | 0.74 (0.47 - 0.87) | 0.77 (0.53 - 0.92) | 0.74 (0.43 - 0.92) |
| HYBRID_GTV | 0.73 +/- 0.06 | 0.76 +/- 0.08 | 0.76 +/- 0.1 | 0.75 +/- 0.08 |
| HYBRID_GTV | 0.73 (0.55 - 0.91) | 0.76 (0.53 - 0.93) | 0.77 (0.49 - 0.95) | 0.75 (0.49 - 0.95) |
| CLINICAL_CTV | 0.85 +/- 0.06 | 0.8 +/- 0.08 | 0.78 +/- 0.09 | 0.81 +/- 0.08 |
| CLINICAL_CTV | 0.87 (0.64 - 0.93) | 0.83 (0.55 - 0.93) | 0.8 (0.57 - 0.95) | 0.84 (0.55 - 0.95) |
| HYBRID_CTV | 0.83 +/- 0.07 | 0.82 +/- 0.06 | 0.78 +/- 0.1 | 0.81 +/- 0.08 |
| HYBRID_CTV | 0.85 (0.62 - 0.93) | 0.82 (0.62 - 0.97) | 0.81 (0.52 - 0.96) | 0.83 (0.52 - 0.97) |
| CLINICAL_PTV | 0.86 +/- 0.05 | 0.81 +/- 0.08 | 0.8 +/- 0.08 | 0.83 +/- 0.08 |
| CLINICAL_PTV | 0.88 (0.74 - 0.94) | 0.83 (0.57 - 0.94) | 0.81 (0.6 - 0.96) | 0.85 (0.57 - 0.96) |
| HYBRID_PTV | 0.84 +/- 0.06 | 0.82 +/- 0.07 | 0.79 +/- 0.09 | 0.82 +/- 0.08 |
| HYBRID_PTV | 0.85 (0.63 - 0.94) | 0.82 (0.6 - 0.97) | 0.81 (0.56 - 0.97) | 0.83 (0.56 - 0.97) |
| PAIRED_GTV | 0.57 +/- 0.08 | 0.57 +/- 0.09 | 0.5 +/- 0.08 | 0.55 +/- 0.09 |
| PAIRED_GTV | 0.55 (0.43 - 0.81) | 0.59 (0.34 - 0.8) | 0.49 (0.42 - 0.74) | 0.54 (0.34 - 0.81) |
| PAIRED_CTV | 0.78 +/- 0.05 | 0.77 +/- 0.07 | 0.69 +/- 0.07 | 0.75 +/- 0.07 |
| PAIRED_CTV | 0.78 (0.66 - 0.86) | 0.75 (0.64 - 0.91) | 0.68 (0.59 - 0.84) | 0.75 (0.59 - 0.91) |
| PAIRED_PTV | 0.8 +/- 0.05 | 0.78 +/- 0.06 | 0.72 +/- 0.06 | 0.77 +/- 0.07 |
| PAIRED_PTV | 0.8 (0.67 - 0.92) | 0.77 (0.66 - 0.89) | 0.71 (0.62 - 0.86) | 0.77 (0.62 - 0.92) |


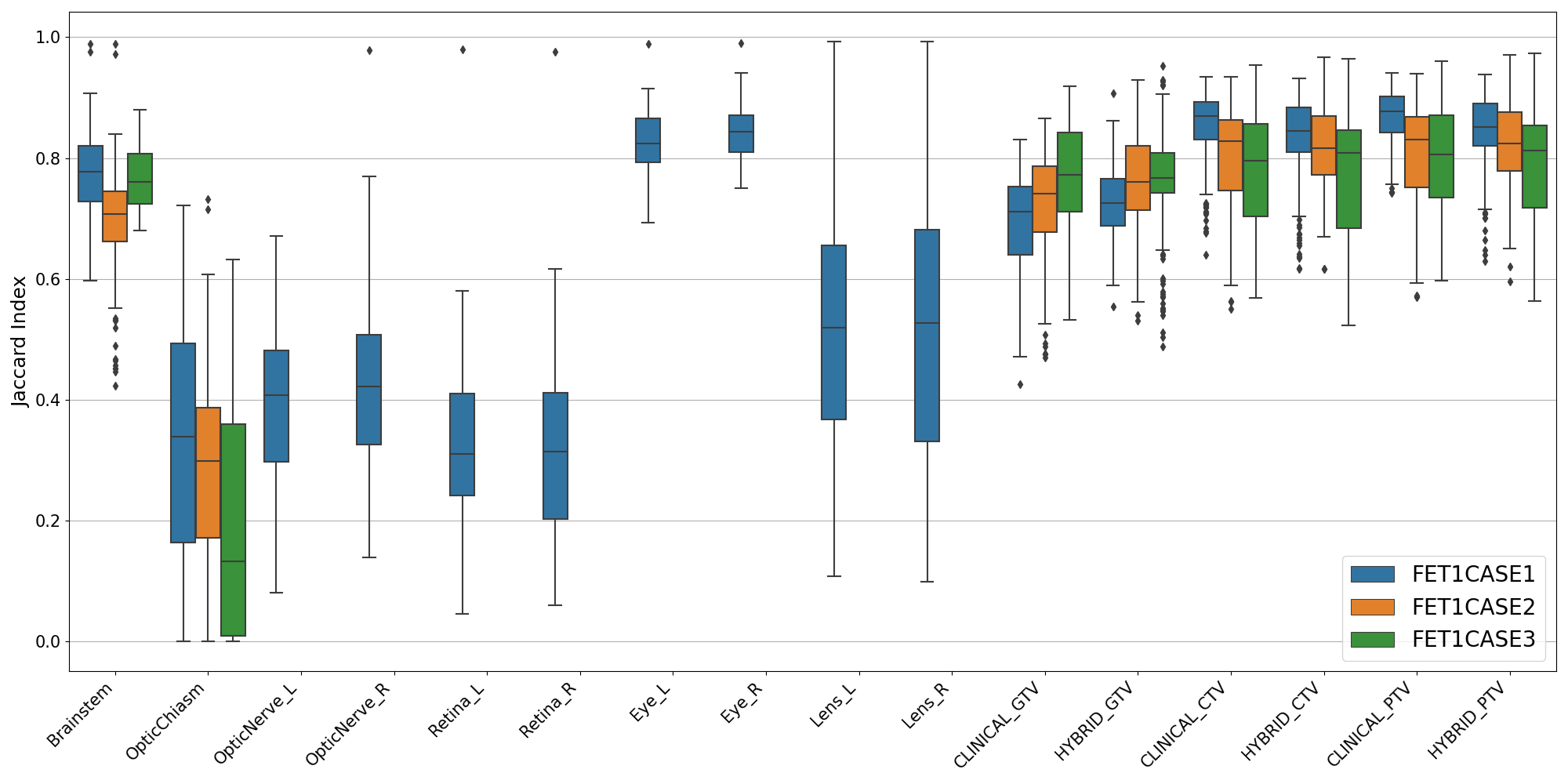


**Supplementary Figure 2.** Boxplots visualising the distribution of pairwise Jaccard Index (JAC) scores for each of the required contours, grouped by the three credentialing cases.

**Supplementary Table 10.** Results of pairwise calculation of overlap volume (OV) to assess spatial overlap of contours delineated for each of the three credentialing cases.

| Structure | FET1CASE1 | FET1CASE2 | FET1CASE3 | ALL |
| --- | --- | --- | --- | --- |
| Brainstem | 0.9 +/- 0.04 | 0.9 +/- 0.05 | 0.91 +/- 0.02 | 0.9 +/- 0.04 |
| Brainstem | 0.91 (0.73 - 1.0) | 0.9 (0.76 - 1.0) | 0.92 (0.85 - 0.95) | 0.91 (0.73 - 1.0) |
| OpticChiasm | 0.53 +/- 0.28 | 0.61 +/- 0.29 | 0.38 +/- 0.31 | 0.53 +/- 0.3 |
| OpticChiasm | 0.62 (0.0 - 0.97) | 0.67 (0.0 - 1.0) | 0.28 (0.0 - 0.99) | 0.61 (0.0 - 1.0) |
| OpticNerve_L | 0.74 +/- 0.11 | None | None | 0.74 +/- 0.11 |
| OpticNerve_L | 0.73 (0.44 - 1.0) | None | None | 0.73 (0.44 - 1.0) |
| OpticNerve_R | 0.71 +/- 0.14 | None | None | 0.71 +/- 0.14 |
| OpticNerve_R | 0.74 (0.31 - 1.0) | None | None | 0.74 (0.31 - 1.0) |
| Retina_L | 0.62 +/- 0.13 | None | None | 0.62 +/- 0.13 |
| Retina_L | 0.62 (0.18 - 1.0) | None | None | 0.62 (0.18 - 1.0) |
| Retina_R | 0.63 +/- 0.14 | None | None | 0.63 +/- 0.14 |
| Retina_R | 0.62 (0.15 - 0.99) | None | None | 0.62 (0.15 - 0.99) |
| Eye_L | 0.95 +/- 0.03 | None | None | 0.95 +/- 0.03 |
| Eye_L | 0.95 (0.88 - 1.0) | None | None | 0.95 (0.88 - 1.0) |
| Eye_R | 0.95 +/- 0.03 | None | None | 0.95 +/- 0.03 |
| Eye_R | 0.95 (0.88 - 1.0) | None | None | 0.95 (0.88 - 1.0) |
| Lens_L | 0.86 +/- 0.11 | None | None | 0.86 +/- 0.11 |
| Lens_L | 0.88 (0.5 - 1.0) | None | None | 0.88 (0.5 - 1.0) |
| Lens_R | 0.86 +/- 0.14 | None | None | 0.86 +/- 0.14 |
| Lens_R | 0.9 (0.38 - 1.0) | None | None | 0.9 (0.38 - 1.0) |
| CLINICAL_GTV | 0.89 +/- 0.06 | 0.93 +/- 0.04 | 0.91 +/- 0.05 | 0.91 +/- 0.05 |
| CLINICAL_GTV | 0.9 (0.72 - 0.99) | 0.94 (0.78 - 0.99) | 0.91 (0.77 - 0.99) | 0.92 (0.72 - 0.99) |
| HYBRID_GTV | 0.89 +/- 0.04 | 0.93 +/- 0.04 | 0.91 +/- 0.05 | 0.91 +/- 0.04 |
| HYBRID_GTV | 0.89 (0.8 - 0.98) | 0.94 (0.81 - 0.99) | 0.92 (0.78 - 1.0) | 0.92 (0.78 - 1.0) |
| CLINICAL_CTV | 0.96 +/- 0.03 | 0.96 +/- 0.03 | 0.94 +/- 0.04 | 0.96 +/- 0.03 |
| CLINICAL_CTV | 0.97 (0.78 - 1.0) | 0.97 (0.82 - 1.0) | 0.95 (0.82 - 1.0) | 0.97 (0.78 - 1.0) |
| HYBRID_CTV | 0.96 +/- 0.02 | 0.96 +/- 0.03 | 0.95 +/- 0.04 | 0.96 +/- 0.03 |
| HYBRID_CTV | 0.96 (0.87 - 1.0) | 0.97 (0.88 - 1.0) | 0.96 (0.79 - 1.0) | 0.96 (0.79 - 1.0) |
| CLINICAL_PTV | 0.97 +/- 0.02 | 0.97 +/- 0.03 | 0.95 +/- 0.04 | 0.97 +/- 0.03 |
| CLINICAL_PTV | 0.98 (0.91 - 1.0) | 0.98 (0.82 - 1.0) | 0.96 (0.82 - 1.0) | 0.97 (0.82 - 1.0) |
| HYBRID_PTV | 0.97 +/- 0.02 | 0.97 +/- 0.03 | 0.96 +/- 0.04 | 0.96 +/- 0.03 |
| HYBRID_PTV | 0.97 (0.88 - 1.0) | 0.97 (0.9 - 1.0) | 0.97 (0.82 - 1.0) | 0.97 (0.82 - 1.0) |
| PAIRED_GTV | 0.96 +/- 0.06 | 0.95 +/- 0.09 | 0.99 +/- 0.01 | 0.97 +/- 0.07 |
| PAIRED_GTV | 0.97 (0.81 - 1.0) | 0.99 (0.58 - 1.0) | 1.0 (0.95 - 1.0) | 0.99 (0.58 - 1.0) |
| PAIRED_CTV | 0.97 +/- 0.04 | 0.98 +/- 0.02 | 0.97 +/- 0.04 | 0.97 +/- 0.03 |
| PAIRED_CTV | 0.98 (0.83 - 1.0) | 0.99 (0.94 - 1.0) | 0.99 (0.81 - 1.0) | 0.98 (0.81 - 1.0) |
| PAIRED_PTV | 0.98 +/- 0.02 | 0.99 +/- 0.02 | 0.98 +/- 0.04 | 0.98 +/- 0.02 |
| PAIRED_PTV | 0.99 (0.93 - 1.0) | 0.99 (0.94 - 1.0) | 0.99 (0.84 - 1.0) | 0.99 (0.84 - 1.0) |


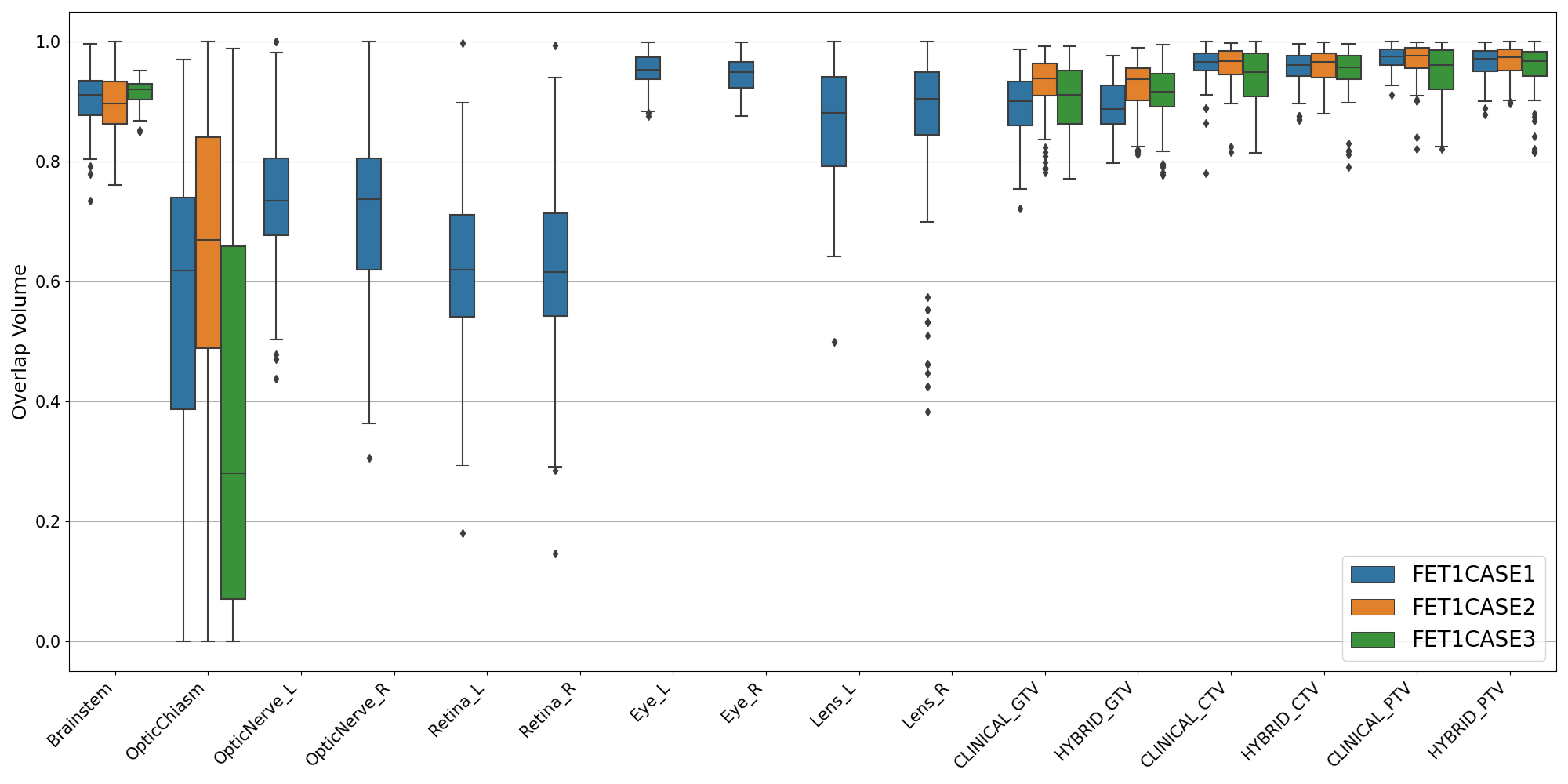


**Supplementary Figure 3.** Boxplots visualising the distribution of pairwise Overlap Volume (OV) scores for each of the required contours, grouped by the three credentialing cases.

**Supplementary Table 11.** Results of pairwise calculation of Hausdorff Distance (HD) to assess boundary differences between all ROs for contours delineated on each of the three credentialing cases.

| Structure | FET1CASE1 | FET1CASE2 | FET1CASE3 | ALL |
| --- | --- | --- | --- | --- |
| Brainstem | 6.92 +/- 1.98 | 11.26 +/- 5.52 | 7.83 +/- 2.64 | 8.66 +/- 4.26 |
| Brainstem | 6.91 (2.5 - 12.5) | 10.0 (1.07 - 31.52) | 7.5 (4.22 - 15.04) | 7.65 (1.07 - 31.52) |
| OpticChiasm | 6.45 +/- 2.65 | 9.08 +/- 4.68 | 13.34 +/- 7.85 | 8.74 +/- 5.39 |
| OpticChiasm | 6.02 (2.4 - 16.63) | 7.77 (2.5 - 18.96) | 10.09 (2.4 - 27.5) | 6.93 (2.4 - 27.5) |
| OpticNerve_L | 7.12 +/- 4.86 | None | None | 7.12 +/- 4.86 |
| OpticNerve_L | 5.37 (2.5 - 22.97) | None | None | 5.37 (2.5 - 22.97) |
| OpticNerve_R | 5.98 +/- 3.69 | None | None | 5.98 +/- 3.69 |
| OpticNerve_R | 4.89 (1.07 - 18.65) | None | None | 4.89 (1.07 - 18.65) |
| Retina_L | 10.45 +/- 3.87 | None | None | 10.45 +/- 3.87 |
| Retina_L | 9.59 (1.07 - 19.61) | None | None | 9.59 (1.07 - 19.61) |
| Retina_R | 10.87 +/- 4.13 | None | None | 10.87 +/- 4.13 |
| Retina_R | 10.77 (1.07 - 19.12) | None | None | 10.77 (1.07 - 19.12) |
| Eye_L | 3.2 +/- 0.66 | None | None | 3.2 +/- 0.66 |
| Eye_L | 3.22 (1.07 - 5.41) | None | None | 3.22 (1.07 - 5.41) |
| Eye_R | 3.17 +/- 0.64 | None | None | 3.17 +/- 0.64 |
| Eye_R | 3.22 (1.07 - 5.0) | None | None | 3.22 (1.07 - 5.0) |
| Lens_L | 2.9 +/- 1.03 | None | None | 2.9 +/- 1.03 |
| Lens_L | 2.5 (1.07 - 5.55) | None | None | 2.5 (1.07 - 5.55) |
| Lens_R | 2.79 +/- 1.08 | None | None | 2.79 +/- 1.08 |
| Lens_R | 2.61 (1.07 - 5.92) | None | None | 2.61 (1.07 - 5.92) |
| CLINICAL_GTV | 10.46 +/- 6.66 | 17.25 +/- 7.94 | 4.44 +/- 2.59 | 11.41 +/- 8.19 |
| CLINICAL_GTV | 7.74 (2.93 - 28.89) | 20.19 (4.22 - 30.64) | 3.47 (2.15 - 13.3) | 7.74 (2.15 - 30.64) |
| HYBRID_GTV | 8.57 +/- 3.18 | 14.33 +/- 6.26 | 4.96 +/- 1.6 | 9.76 +/- 5.76 |
| HYBRID_GTV | 8.39 (3.04 - 18.56) | 13.05 (3.3 - 26.65) | 4.97 (2.15 - 10.0) | 8.23 (2.15 - 26.65) |
| CLINICAL_CTV | 11.08 +/- 4.92 | 14.74 +/- 5.53 | 8.83 +/- 4.38 | 11.85 +/- 5.56 |
| CLINICAL_CTV | 9.22 (3.22 - 21.61) | 14.82 (4.08 - 24.97) | 8.08 (2.5 - 18.18) | 10.99 (2.5 - 24.97) |
| HYBRID_CTV | 9.01 +/- 3.01 | 13.26 +/- 5.29 | 9.77 +/- 4.11 | 10.78 +/- 4.67 |
| HYBRID_CTV | 8.75 (4.22 - 16.73) | 11.9 (3.22 - 26.54) | 9.37 (2.72 - 17.9) | 10.06 (2.72 - 26.54) |
| CLINICAL_PTV | 11.42 +/- 4.56 | 14.93 +/- 5.62 | 8.99 +/- 4.2 | 12.09 +/- 5.44 |
| CLINICAL_PTV | 10.39 (2.93 - 21.92) | 15.26 (4.22 - 25.4) | 8.95 (2.5 - 17.47) | 11.8 (2.5 - 25.4) |
| HYBRID_PTV | 9.15 +/- 3.35 | 13.55 +/- 5.43 | 10.78 +/- 4.16 | 11.2 +/- 4.82 |
| HYBRID_PTV | 9.01 (3.4 - 17.7) | 12.5 (3.3 - 27.04) | 11.82 (2.72 - 18.43) | 10.63 (2.72 - 27.04) |
| PAIRED_GTV | 15.54 +/- 1.76 | 13.26 +/- 3.25 | 8.83 +/- 1.12 | 12.75 +/- 3.54 |
| PAIRED_GTV | 15.59 (11.94 - 18.31) | 12.14 (10.28 - 24.6) | 8.81 (6.93 - 10.99) | 12.21 (6.93 - 24.6) |
| PAIRED_CTV | 14.93 +/- 1.88 | 12.25 +/- 3.29 | 9.09 +/- 2.09 | 12.26 +/- 3.44 |
| PAIRED_CTV | 14.66 (11.34 - 18.56) | 11.9 (8.43 - 23.72) | 8.81 (5.37 - 14.82) | 12.11 (5.37 - 23.72) |
| PAIRED_PTV | 14.81 +/- 2.06 | 12.39 +/- 3.19 | 9.33 +/- 2.46 | 12.33 +/- 3.42 |
| PAIRED_PTV | 14.63 (10.44 - 18.56) | 12.19 (8.43 - 23.72) | 8.86 (4.8 - 15.64) | 12.3 (4.8 - 23.72) |


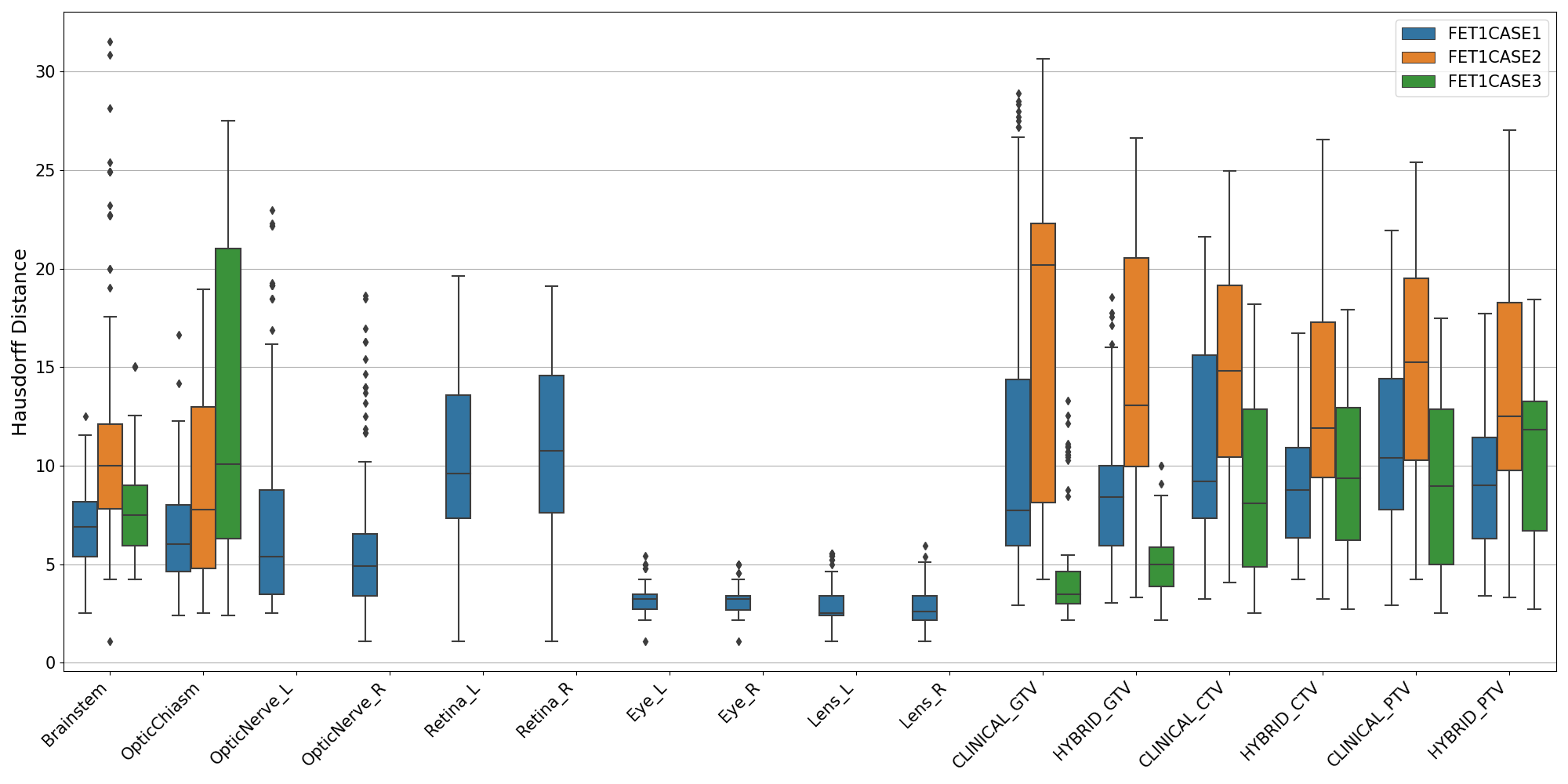


**Supplementary Figure 4.** Boxplots visualising the distribution of pairwise Hausdorff Distance (HD) for each of the required contours, grouped by the three credentialing cases.

**Supplementary Table 12.** Results of the pairwise calculation of Mean Absolute Surface Distance (MASD) to assess boundary differences between all ROs for contours delineated on each of the three credentialing cases.

| Structure | FET1CASE1 | FET1CASE2 | FET1CASE3 | ALL |
| --- | --- | --- | --- | --- |
| Brainstem | 0.99 +/- 0.31 | 1.48 +/- 0.75 | 1.11 +/- 0.34 | 1.19 +/- 0.57 |
| Brainstem | 1.0 (0.05 - 1.76) | 1.29 (0.02 - 4.35) | 1.1 (0.4 - 1.69) | 1.1 (0.02 - 4.35) |
| OpticChiasm | 1.94 +/- 1.9 | 2.15 +/- 1.74 | 3.26 +/- 1.87 | 2.27 +/- 1.9 |
| OpticChiasm | 1.29 (0.28 - 9.55) | 1.62 (0.27 - 9.52) | 3.59 (0.31 - 6.99) | 1.56 (0.27 - 9.55) |
| OpticNerve_L | 1.41 +/- 1.18 | None | None | 1.41 +/- 1.18 |
| OpticNerve_L | 0.94 (0.39 - 5.48) | None | None | 0.94 (0.39 - 5.48) |
| OpticNerve_R | 1.06 +/- 0.64 | None | None | 1.06 +/- 0.64 |
| OpticNerve_R | 0.91 (0.02 - 3.67) | None | None | 0.91 (0.02 - 3.67) |
| Retina_L | 1.89 +/- 1.1 | None | None | 1.89 +/- 1.1 |
| Retina_L | 1.55 (0.02 - 5.46) | None | None | 1.55 (0.02 - 5.46) |
| Retina_R | 2.21 +/- 1.6 | None | None | 2.21 +/- 1.6 |
| Retina_R | 1.59 (0.02 - 6.38) | None | None | 1.59 (0.02 - 6.38) |
| Eye_L | 0.58 +/- 0.21 | None | None | 0.58 +/- 0.21 |
| Eye_L | 0.58 (0.02 - 1.24) | None | None | 0.58 (0.02 - 1.24) |
| Eye_R | 0.51 +/- 0.16 | None | None | 0.51 +/- 0.16 |
| Eye_R | 0.5 (0.03 - 0.87) | None | None | 0.5 (0.03 - 0.87) |
| Lens_L | 0.88 +/- 0.53 | None | None | 0.88 +/- 0.53 |
| Lens_L | 0.69 (0.02 - 2.41) | None | None | 0.69 (0.02 - 2.41) |
| Lens_R | 0.83 +/- 0.55 | None | None | 0.83 +/- 0.55 |
| Lens_R | 0.73 (0.03 - 2.93) | None | None | 0.73 (0.03 - 2.93) |
| CLINICAL_GTV | 1.11 +/- 0.69 | 2.07 +/- 1.06 | 0.86 +/- 0.46 | 1.4 +/- 0.96 |
| CLINICAL_GTV | 0.93 (0.34 - 3.76) | 1.85 (0.58 - 5.16) | 0.78 (0.18 - 2.43) | 1.07 (0.18 - 5.16) |
| HYBRID_GTV | 1.1 +/- 0.36 | 1.69 +/- 0.69 | 1.06 +/- 0.56 | 1.31 +/- 0.62 |
| HYBRID_GTV | 1.07 (0.22 - 2.32) | 1.7 (0.34 - 3.67) | 1.01 (0.16 - 2.66) | 1.16 (0.16 - 3.67) |
| CLINICAL_CTV | 1.24 +/- 0.72 | 2.06 +/- 1.16 | 1.5 +/- 0.83 | 1.61 +/- 1.0 |
| CLINICAL_CTV | 1.02 (0.39 - 3.89) | 1.64 (0.43 - 5.91) | 1.25 (0.18 - 4.09) | 1.28 (0.18 - 5.91) |
| HYBRID_CTV | 1.48 +/- 0.82 | 1.98 +/- 0.86 | 1.72 +/- 0.96 | 1.73 +/- 0.9 |
| HYBRID_CTV | 1.32 (0.44 - 4.37) | 1.97 (0.24 - 5.2) | 1.38 (0.19 - 4.21) | 1.5 (0.19 - 5.2) |
| CLINICAL_PTV | 1.26 +/- 0.6 | 2.27 +/- 1.27 | 1.65 +/- 0.9 | 1.74 +/- 1.06 |
| CLINICAL_PTV | 1.1 (0.39 - 2.86) | 1.79 (0.43 - 6.29) | 1.4 (0.19 - 4.39) | 1.41 (0.19 - 6.29) |
| HYBRID_PTV | 1.61 +/- 0.85 | 2.19 +/- 1.05 | 1.95 +/- 1.03 | 1.91 +/- 1.01 |
| HYBRID_PTV | 1.52 (0.45 - 4.77) | 2.08 (0.23 - 6.12) | 1.63 (0.16 - 5.0) | 1.73 (0.16 - 6.12) |
| PAIRED_GTV | 2.22 +/- 0.52 | 3.09 +/- 0.84 | 2.86 +/- 0.63 | 2.72 +/- 0.78 |
| PAIRED_GTV | 2.31 (0.86 - 3.0) | 3.05 (1.06 - 4.9) | 2.91 (1.17 - 3.96) | 2.82 (0.86 - 4.9) |
| PAIRED_CTV | 2.34 +/- 0.59 | 2.57 +/- 0.89 | 2.49 +/- 0.75 | 2.47 +/- 0.76 |
| PAIRED_CTV | 2.27 (1.26 - 3.74) | 2.7 (0.88 - 4.53) | 2.78 (0.9 - 3.54) | 2.49 (0.88 - 4.53) |
| PAIRED_PTV | 2.38 +/- 0.71 | 2.66 +/- 0.88 | 2.68 +/- 0.73 | 2.57 +/- 0.79 |
| PAIRED_PTV | 2.37 (0.83 - 4.1) | 2.82 (1.17 - 4.71) | 2.89 (1.01 - 3.8) | 2.49 (0.83 - 4.71) |


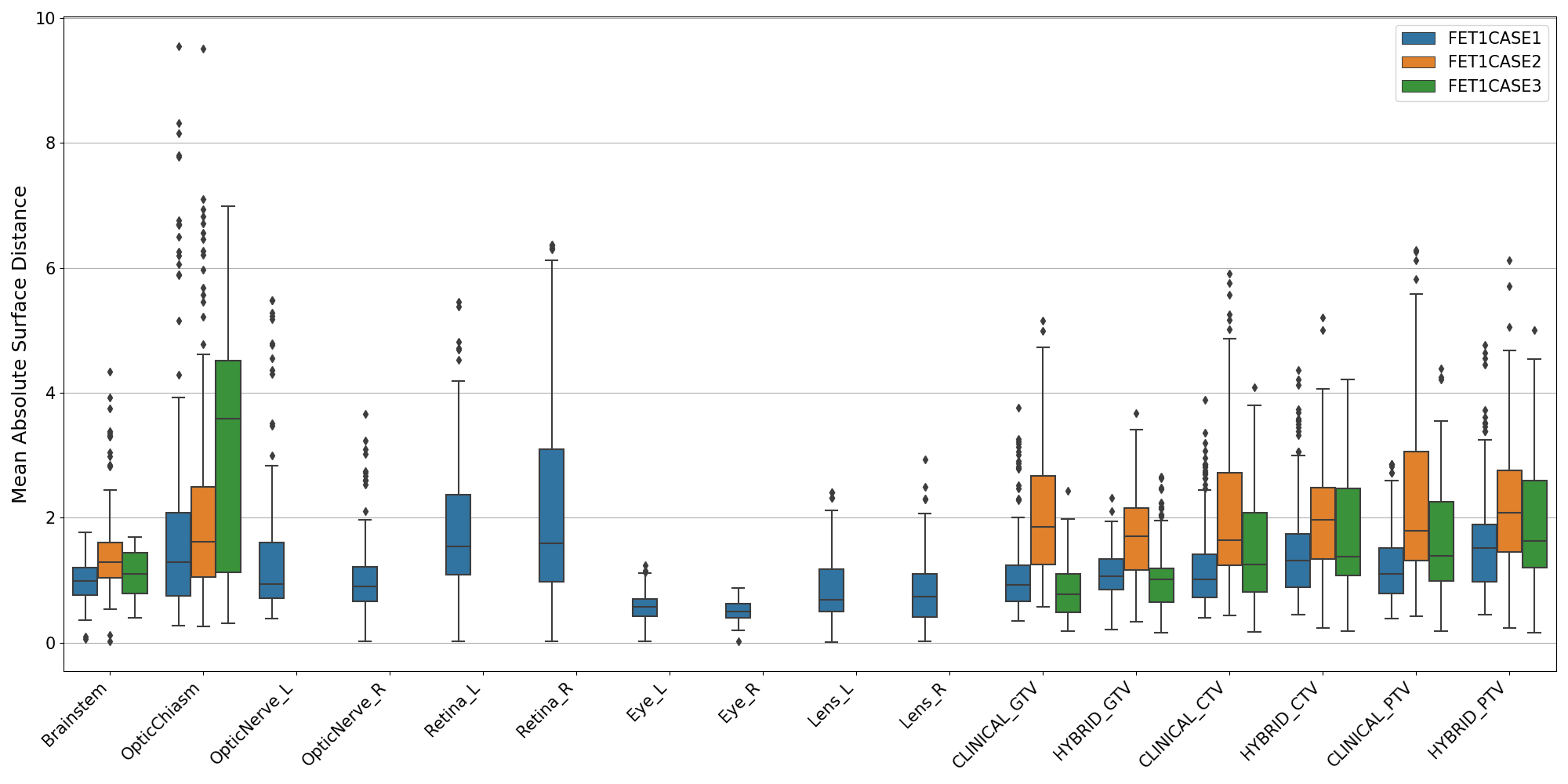


**Supplementary Figure 5.** Boxplots visualising the distribution of pairwise Mean Absolute Surface Distance (MASD) for each of the required contours, grouped by the three credentialing cases.


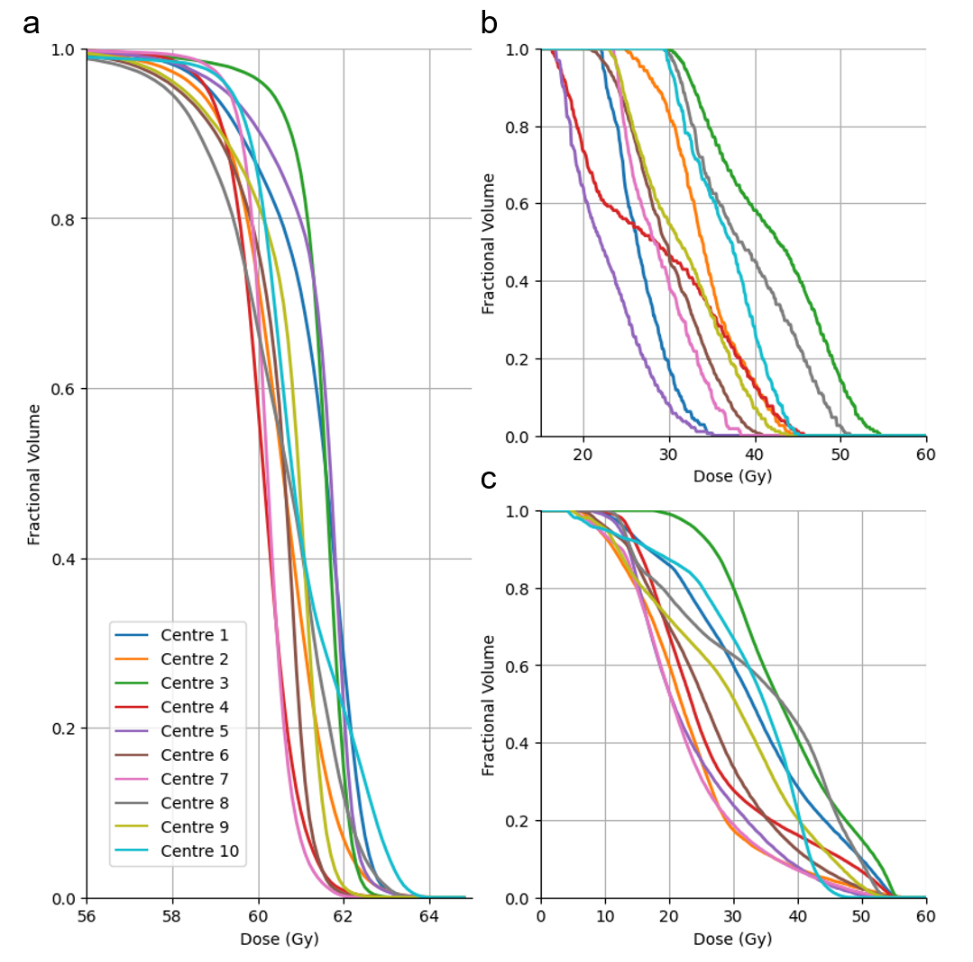


**Supplementary Figure 6.** Dose volume histograms of (a) PTV (b) Optic Chiasm and (c) Brainstem from all 10 centres. Each centre was instructed to develop a dose map on a single planning case with standard-of-care pre-generated contours. All plans were within dose constraints.

**Supplementary Table 13.** Ten radiotherapy plans were received as part of trial site credentialing. A series of dose metrics were extracted from each to evaluate variability across sites, as shown below, for OARs and PTV.

|  | **D_98%_ (Gy)** | **D_50%_ (Gy)** | **D_2%_ (Gy)** |
| --- | --- | --- | --- |
| Brainstem | 9.57 (5.85-21.54) | 28.1 (20.59-37.75) | 50.75 (45.01-54.72) |
| Optic Chiasm | 23.55 (16.67-30.97) | 30.41 (22.0-42.7) | 42.72 (33.02-53.43) |
| PTV | 58.13 (56.77-59.18) | 60.75 (60.13-61.7) | 62.50 (61.46-63.33) |
| Left Optic Nerve | 7.93 (3.12-11.99) | 12.84 (7.71-25.2) | 22.94 (11.5-33.72) |
| Right Optic Nerve | 12.01 (3.93-17.95) | 26.48 (17.0-29.12) | 40.67 (26.28-47.35) |
| Left Retina | 1.33 (1.12-1.7) | 3.03 (2.2-5.08) | 8.79 (3.78-15.68) |
| Right Retina | 1.75 (1.34-3.56) | 3.82 (3.0-9.37) | 12.52 (3.89-20.45) |
| Left Lens | 1.43 (1.14-1.94) | 1.9 (1.37-2.62) | 2.35 (1.69-3.5) |
| Right Lens | 1.76 (1.32-4.03) | 2.12 (1.62-4.9) | 2.67 (2.19-7.4) |
| Left Eye | 1.22 (1.0-1.76) | 3.2 (1.97-4.77) | 8.51 (3.64-14.91) |
| Right Eye | 1.52 (1.3-3.48) | 3.19 (2.58-7.46) | 11.47 (3.68-19.63) |
|  | **V_95%_ (cc)** | **CI** | **HI** |
| PTV | 322.95 (318.8- 324.5) | 0.99 (0.98-1.0) | 7.61 (4.4-10.14) |
| Median (range) |  |  |  |
